# Ethosuximide ameliorates neurodegenerative disease phenotypes by modulating DAF-16/FOXO target gene expression

**DOI:** 10.1186/s13024-015-0046-3

**Published:** 2015-09-29

**Authors:** Xi Chen, Hannah V. McCueF, Shi Quan Wong, Sudhanva S. Kashyap, Brian C. Kraemer, Jeff W. Barclay, Robert D. Burgoyne, Alan Morgan

**Affiliations:** Department of Cellular and Molecular Physiology, Institute of Translational Medicine, University of Liverpool, Crown St, Liverpool, L69 3BX UK; Geriatrics Research Education and Clinical Center, Seattle Veterans Affairs Puget Sound Health Care System and University of Washington Department of Medicine, 1660 South Columbian Way, Seattle, WA 98108 USA; Present Address: Centre for Neurodegenerative Science, Van Andel Research Institute, 333 Bostwick Avenue NE, Grand Rapids, MI 49503 USA

**Keywords:** Adult onset neuronal lipofuscinosis, Frontotemporal dementia, Neurodegeneration, Aging, Caenorhabditis elegans, Neuroblastoma

## Abstract

**Background:**

Many neurodegenerative diseases are associated with protein misfolding/aggregation. Treatments mitigating the effects of such common pathological processes, rather than disease-specific symptoms, therefore have general therapeutic potential.

**Results:**

Here we report that the anti-epileptic drug ethosuximide rescues the short lifespan and chemosensory defects exhibited by *C. elegans* null mutants of *dnj-14*, the worm orthologue of the *DNAJC5* gene mutated in autosomal-dominant adult-onset neuronal ceroid lipofuscinosis. It also ameliorates the locomotion impairment and short lifespan of worms expressing a human Tau mutant that causes frontotemporal dementia. Transcriptomic analysis revealed a highly significant up-regulation of DAF-16/FOXO target genes in response to ethosuximide; and indeed RNAi knockdown of *daf-16* abolished the therapeutic effect of ethosuximide in the worm *dnj-14* model. Importantly, ethosuximide also increased the expression of classical FOXO target genes and reduced protein aggregation in mammalian neuronal cells.

**Conclusions:**

We have revealed a conserved neuroprotective mechanism of action of ethosuximide from worms to mammalian neurons. Future experiments in mouse neurodegeneration models will be important to confirm the repurposing potential of this well-established anti-epileptic drug for treatment of human neurodegenerative diseases.

**Electronic supplementary material:**

The online version of this article (doi:10.1186/s13024-015-0046-3) contains supplementary material, which is available to authorized users.

## Background

A major challenge in current neurodegeneration research is the identification of effective therapies. Over recent years, simple model organisms, such as the nematode worm *Caenorhabditis elegans*, have been increasingly recognised as powerful systems for revealing the conserved molecular mechanisms that underlie neurodegeneration [1]. Indeed various laboratories have developed and characterised a diverse set of *C. elegans* models of various human neurodegenerative diseases, including Alzheimer’s [2], Parkinson’s [3] and polyglutamine expansion diseases [4]. Genetic screens performed in these models have identified a variety of genes that can suppress or increase disease progression and are thus potential therapeutic drug targets. However, relatively few of these genetic modifiers are common to more than one disease model, despite the shared feature of protein misfolding/aggregation [5, 6].

Complementary to its utility for genetic screens, *C. elegans* is a useful pharmacological model for testing potential neuroprotective compounds. Attention has mainly focused on screening existing FDA-approved medications rather than novel compounds, as repurposing of drugs pre-approved for other indications obviates the need for early toxicity trials and thus expedites translation to clinical testing [7, 8]. For example, *C. elegans* Alzheimer’s models expressing human Aβ_1–42_ have identified neuroprotective effects of several approved compounds, including antibiotic, antidepressant and antihypertensive drugs [9]. Similarly, dopamine D2 receptor antagonists have been shown to ameliorate mutant tau-induced functional defects and reduce aggregation in a frontotemporal dementia with parkinsonism-17 (FTDP-17) tauopathy model [10]. A wide variety of other neuroprotective compounds have also been identified in chemical screens using worm neurodegeneration models including spinal muscular atrophy [11], Parkinson’s [12] and Huntington’s diseases [13].

Most compounds identified in *C. elegans* chemical screens to date are effective in only a single neurodegenerative model, suggesting that translational potential may be disease-specific. However, some compounds, such as resveratrol, have been shown to be protective in a range of worm models and also in mammalian systems [14–18]. This demonstrates that it is possible to identify generally neuroprotective compounds that alleviate the functional consequences of protein misfolding common to neurodegeneration. Here we report that ethosuximide, a widely prescribed anti-epileptic drug, improves the phenotypes of multiple neurodegenerative disease models and we reveal a conserved action of the drug in modulating DAF-16/FOXO target gene expression in worms and mammalian neurons.

## Results

### Ethosuximide ameliorates *C. elegans dnj-14* mutant phenotypes

**Fig. 1:**
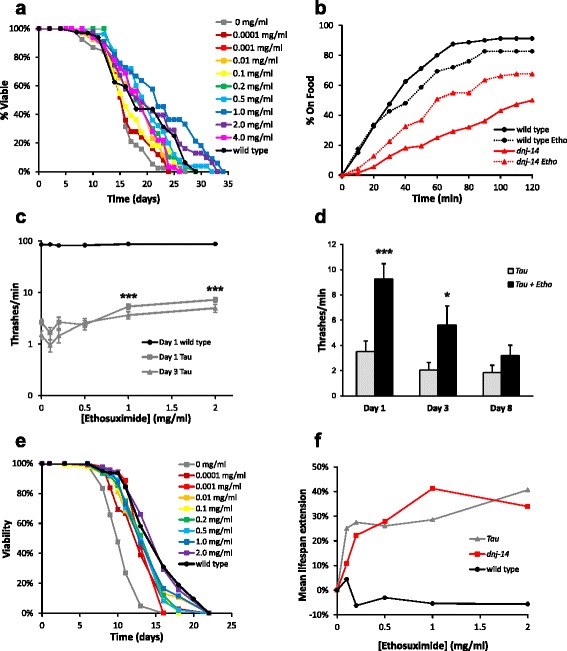
Ethosuximide increases lifespan and improves sensorimotor function in *C. elegans* ANCL and frontotemporal dementia models*.* **a** Ethosuximide extends lifespan in *dnj-14* mutants. Viability of age-synchronised *dnj-14(ok237)* animals grown in the presence of the indicated concentrations of ethosuximide was determined; untreated wild type control N2 worms are shown for comparison (n = 50-55 worms for each concentration). **b** Ethosuximide ameliorates the *dnj-14* food sensing defect. The time taken to move to a bacterial food source was measured in wild type N2 and *dnj-14(tm3223)* strains grown until 5-6 days of age in the presence or absence of ethosuximide (n = 71-80 worms of each strain per condition). **c** Ethosuximide increases locomotion in Tau V337M worms, but not control worms. Thrashing in solution was measured in Tau V337M worms grown until 1 and 3 days of age and assayed in the presence of the indicated concentrations of ethosuximide (for each age group, n = 120-140 worms for 0 mg/l; n = 38-40 worms for 0.1, 0.2 and 0.5 mg/ml; n = 80-90 worms for 1 and 2 mg/ml;). Identically treated wild type control CZ1200 worms are shown for comparison (n = 20 worms per concentration). Data are shown as mean ± SEM (***p < 0.001). **d** Age-dependence of ethosuximide’s effect on Tau V337M locomotion. Thrashing assays were performed on age-synchronised animals grown in the presence or absence of 2 mg/ml ethosuximide (n = 30-50 worms per data point). Data are shown as mean ± SEM (***p < 0.001, *p < 0.05). **e** Ethosuximide increases lifespan in Tau V337M worms. Viability of age-synchronised animals grown in the presence of the indicated concentrations of ethosuximide was determined in comparison to untreated wild type control CZ1200 worms (n = 50-102 worms for each drug concentration). **f** Comparison of the ethosuximide concentration-dependence of mean lifespan extension in *dnj-14* and Tau V337M worms

The rare hereditary human neurodegenerative disease, autosomal-dominant adult-onset neuronal ceroid lipofuscinosis (ANCL), is caused by mutations in the *DNAJC5* gene [19–22]. *DNAJC5* encodes a neuronal chaperone of the DnaJ/Hsp40 family of molecular chaperones known as cysteine string protein (CSP), which prevents the misfolding of presynaptic proteins [23–27]. DNJ-14 is the worm orthologue of CSP and *dnj-14* null mutants are characterised by reduced lifespan and age-dependent sensorimotor defects and neurodegeneration, similar to CSP knockout mice [18, 28]. We used this *dnj-14* model to screen for compounds with therapeutic potential for ANCL and possibly other neurodegenerative diseases, by testing their ability to extend the short lifespan of *dnj-14(ok237)* worms [18]. The anti-epileptic drug, ethosuximide, was observed to produce a robust and reproducible lifespan extension in *dnj-14(ok237)* animals. This effect was concentration-dependent, with 1 mg/ml ethosuximide offering the most significant lifespan increase, raising the mean lifespan of *dnj-14(ok237)* worms by over 40 % (Fig. 1a) Over a series of experiments, this optimal concentration produced a near-complete rescue of lifespan in dnj-14 mutants to levels close to that of wild-type N2 worms (Additional file 1: Table S1). At the highest concentration used (4 mg/ml), ethosuximide produced no significant increase in lifespan. Notably, none of the concentrations used had any significant effect on the lifespan of wild-type N2 *C. elegans* (Additional file 2: Figure S1A; Additional file 1: Table S1). To test if ethosuximide was also able to rescue the sensory defect in *dnj-14* mutants, we performed a food race assay, which measures the time taken for animals to move a defined distance to a bacterial food source (Fig. 1b). As previously observed [18], *dnj-14* mutants are severely impaired in this assay. Ethosuximide significantly improved food sensing activity of *dnj-14* mutants, approximately doubling the number of worms reaching the food within 60 min, although complete rescue to wild type levels was not achieved (Fig. 1b). Ethosuximide had no stimulatory activity in food race assays using wild type N2 worms (Fig. 1b), nor did it increase locomotion of *dnj-14* or N2 worms in thrashing assays (Additional file 2: Figure S1B). Therefore the stimulatory action of ethosuximide in food race assays appears to be due to a specific effect on the chemosensory defect in *dnj-14* mutants rather than a generic stimulation of movement. Taken together, these data suggest that ethosuximide is able to ameliorate the neurotoxicity induced by the loss of the DNJ-14 synaptic chaperone protein.

### Ethosuximide alleviates phenotypes caused by expression of human mutant Tau

To determine if ethosuximide had general neuroprotective activity, we evaluated its effects on a *C. elegans* frontotemporal dementia with parkinsonism-17 (FTDP-17) tauopathy model [29]. FTDP-17 is one of many human tauopathies in which characteristic neurofibrillary tangles are formed from hyperphosphorylated Tau. Overexpression of human mutant Tau V337M throughout the *C. elegans* nervous system causes severe motility defects, neurodegeneration, short lifespan and accumulation of insoluble Tau [29]. The highly penetrant and easily observable motility phenotype of this model is well suited for assessing drug effects [10]. We observed that ethosuximide improved the severely uncoordinated phenotype of Tau V337M transgenic worms in solution. To quantify this effect, we performed thrashing assays in the presence of varying concentrations of ethosuximide. As shown in Fig. 1c, ethosuximide increased the thrashing frequency of young worms in a dose-dependent manner, with an optimal concentration of 1-2 mg/ml. Although this stimulatory effect was highly significant (*P* < 0.001) and approximately doubled thrashing rates at these concentrations, this still represents only a relatively small increase that is far from a complete rescue to wild type levels. Nevertheless, the effects were Tau-specific, because control worms showed wild-type thrashing activity that was not increased by ethosuximide (Fig. 1c; Additional file 2: Figure S1C). Ethosuximide significantly increased thrashing in young animals (day 1 and 3), but its therapeutic activity declined in older animals (Fig. 1d). To directly test the effect of ethosuximide on longevity, lifespan assays were performed on ethosuximide treated Tau V337M and wild type worms and compared with vehicle-treated controls. Ethosuximide significantly enhanced the mean lifespan of Tau V337M worms in a concentration-dependent manner (Fig. 1e; Additional file 1: Table S1), but had no effect on wild type control worms (Additional file 2: Figure S1D; Additional file 1: Table S1). Maximal lifespan extension was seen at 2 mg/ml, which conferred a 40 % lifespan increase - comparable to the longevity effect of ethosuximide seen with *dnj-14* mutants – although wild type lifespan was not significantly affected at any concentration (Fig. 1f).

### Ethosuximide action is independent of T-type calcium channels

**Fig. 2:**
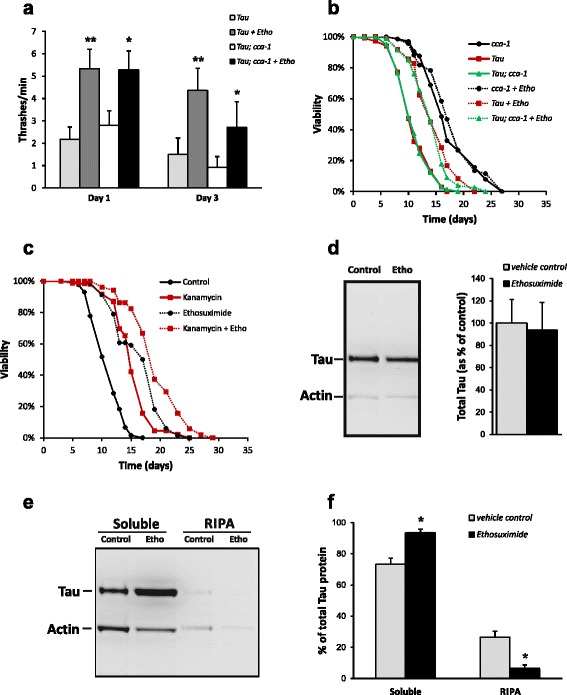
Ethosuximide acts independently of T-type calcium channels and bacterial metabolism to reduce Tau aggregation. **a** Ethosuximide Inhibition of the T-type calcium channel, CCA-1, is not required for protection against paralysis. Tau V337M transgenic animals were crossed with loss-of-function mutations for *cca-1(ad1650)* to generate homozygous cross progeny. Ethosuximide supplementation increased thrashing activity of the Tau V337M transgenic strain and the double mutant Tau V337M; *cca-1(ad1650)* strain to similar extents. Data are shown as mean ± SEM (**p < 0.01, *p < 0.05; n = 30-50 worms per data point). **b** Ethosuximide extends lifespan in Tau V337M mutants in the absence of CCA-1. Lifespan assays were performed on single mutant *cca-1(ad1650)*, transgenic (Tau V337M) and double mutant Tau V337M; *cca-1(ad1650)* strains grown in the presence (dashed lines) or absence (solid lines) of 1 mg/ml ethosuximide (n > 100 worms per strain/condition). **c** Ethosuximide extends lifespan using killed bacteria as a food source. Lifespan assays were performed on Tau V337M worms grown under control conditions or in the presence of kanamycin, or 1 mg/ml ethosuximide or both (n > 80 worms per condition). **d** Total Tau protein expression in Tau V337M worm lysates is not reduced by ethosuximide. The left panel shows a representative western blot; the right panel shows quantification of Tau normalised to actin and expressed as % of untreated control (mean ± SEM, n = 3; not significant). **e**, **f** Ethosuximide affects Tau proteostasis. **e** shows a representative western blot of the soluble and detergent-soluble (RIPA) sequentially extracted fractions in the presence or absence of ethosuximide treatment. **f** shows quantification of Tau fractions normalised to actin and expressed as % of the total (soluble + RIPA) protein level (data are shown as mean ± SEM (n = 3; *p < 0.05)

The efficacy of ethosuximide in generalised absence epilepsy is thought to be due to blockade of the low voltage activated T-type calcium channel [30]. *C. elegans* CCA-1 is most similar to the vertebrate T-type calcium channel α1 subunit (42 % identity), with typical T-type kinetics, voltage dependence and pharmacology [31]. We therefore constructed a double mutant Tau V337M; *cca-1(ad1650)* strain to determine if ethosuximide’s therapeutic action in the frontotemporal dementia model was mediated via inhibition of CCA-1. We chose to use the Tau model because it is extremely challenging technically to cross *cca-1* with *dnj-14* mutants, as both genes are located on the X chromosome. As seen in Fig. 2a, loss of *cca-1* had minimal effect on Tau proteotoxicity, as a similar large percentage of both Tau V337M; *cca-1(ad1650)* homozygotes and Tau V337M transgenic worms exhibited severely impaired motility. In contrast, both *cca-1(ad1650)* single mutant control and heterozygous cross progeny did not exhibit any motility defects. Ethosuximide treatment mitigated the impaired motility of Tau V337M transgenic worms and double mutants harboring a loss-of-function mutation of *cca-1* to a similar extent, both at day 1 and day 3 (Fig. 2a). Tau V337M; *cca-1(ad1650)* double mutants and Tau V337M transgenic worms displayed a mean adult lifespan of 12.1 and 12.6 days, respectively. Following ethosuximide supplementation, there was negligible change in lifespan of *cca-1(ad1650)* single mutant control worms. However, the ability of ethosuximide to increase longevity in the single Tau V337M transgenic strain was maintained in the Tau V337M; *cca-1(ad1650)* homozygotes, significantly extending the mean lifespan by 25 % and 16 %, respectively (Fig. 2b). Taken together, these results indicate that the mechanism of ethosuximide action does not involve inhibition of CCA-1.

### Protective effects of ethosuximide are not due to changes in the *E. coli* food source

One contributor to late-age mortality in *C. elegans* is the detrimental effect of their *E. coli* food source, and drugs that decrease bacterial pathogenicity extend worm lifespan [32]. In addition, it has recently been shown that some drugs, for example metformin, increase *C. elegans* lifespan indirectly via changes to *E. coli* metabolism [33]. To determine if such bacterial effects contribute to the mechanism of action of ethosuximide in lifespan extension, we used the antibiotic kanamycin to kill the OP50 food source and thus prevent both bacterial metabolism and pathogenicity. In single treatments, kanamycin and ethosuximide increased the mean lifespan of Tau V337M worms by 37 % and 48 %, respectively (Fig. 2c). A combined treatment of kanamycin and ethosuximide caused a significant and approximately additive 72 % extension in mean lifespan. As kanamycin clearly did not affect the ability of ethosuximide to increase lifespan (Fig. 2c; Additional file 1: Table S1), this suggests that ethosuximide’s protective effects are independent of bacterial metabolism and pathogenicity.

### Ethosuximide affects Tau protein solubility

In view of the general association between protein misfolding/aggregation and neurodegeneration, we reasoned that ethosuximide’s therapeutic activity might be linked to improved proteostasis. We therefore tested whether ethosuximide could influence the aggregation of mutant Tau protein in the worm frontotemporal dementia model. As shown in Fig. 2d, there was no reduction in total Tau levels in ethosuximide treated Tau transgenic worms as compared to vehicle controls. The rescuing effect of ethosuximide is therefore not due to transgene suppression or reduced expression of toxic mutant Tau protein. We then subjected both vehicle- and ethosuximide-treated Tau V33M transgenic worms to a regimen of sequential extractions with buffers of increasing solubilising strengths, as previously described [29]. Quantification of the amount of soluble and insoluble (RIPA-extractable) Tau relative to total Tau levels revealed a significant reduction in aberrantly-folded, insoluble Tau and a corresponding increase in soluble Tau in ethosuximide-treated compared with untreated worms (Fig. 2e,f). Therefore, ethosuximide’s protective effects on motility and longevity are accompanied by improved Tau proteostasis.

### Ethosuximide modulates expression of DAF-16 target genes

To gain insight into ethosuximide’s mechanism of action, we took an unbiased transcriptomic approach using whole genome *C. elegans* DNA microarrays. Two control strains (N2 and CZ1200) and two ANCL model strains (*dnj-14 ok237* and *tm3223* alleles) were age-synchronised and treated with 1 mg/ml ethosuximide or vehicle control. Gene expression profiling was then used to identify ethosuximide-responsive differentially expressed genes (DEGs) in 6-day-old animals (Additional file 3: Figure S2). Principal component analysis confirmed tight grouping of the triplicate biological samples for each strain and a consistent effect of ethosuximide, as illustrated in the array correlation heatmap (Additional file 4: Figure S3A-C). In order to stringently select for the most consistent and significant ethosuximide-regulated transcripts, we focused on DEGs common to at least 3 out of 4 strains using a 1 % false discovery rate (FDR) cut-off. This yielded 125 DEGs, comprising 61 up-regulated and 64 down-regulated genes. Restricting our analysis still further to transcriptional changes common to all four strains yielded 60 DEGs containing 40 up-regulated and 20 down-regulated genes, as illustrated in Additional file 4: Figure S3D,E. The complete data are shown in Additional file 5: Dataset S1.

**Fig. 3:**
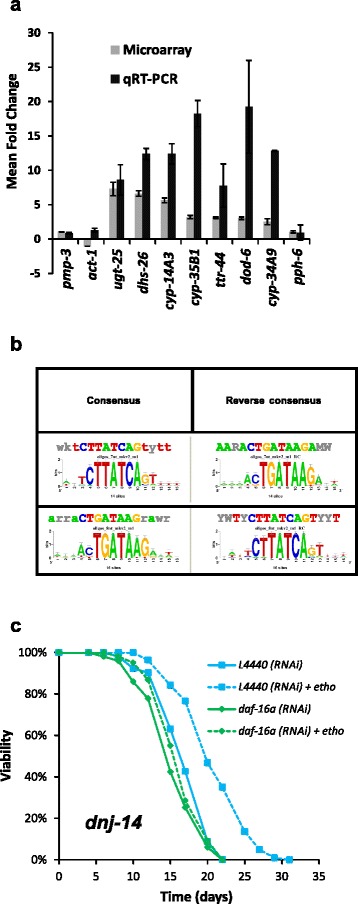
Ethosuximide-induced genes are enriched in DAF-16-associated elements and ethosuximide-induced lifespan extension requires *daf-16*. **a** Validation of gene expression changes using qRT-PCR. Selected genes that were up-regulated by ethosuximide in microarray experiments (*ugt-25, dhs-26, cyp-14A3, cyp-35B1, ttr-44, dod-6* and *cyp-34A9*) were confirmed to be significantly induced using qRT-PCR. No significant changes in expression of normalisation (*act-1*, *pmp-3*) or negative control (*pph-6*) genes was observed. Results are expressed as mean fold change ± SEM relative to the unexposed control (n = 3). **b** Ethosuximide-responsive genes are enriched for the DAF-16 Associated Element (DAE) motif. To identify regulatory sequences correlating with ethosuximide-responsiveness, 200-bp regions in the upstream promoter sequences of common DEGs were mined for overrepresented motifs using RSAT. **c** Ethosuximide increases *dnj-14* lifespan in a *daf-16-*dependent manner. Survival curves of *dnj-14(ok237)* worms grown on *E. coli* containing empty vector (L4440), *hsp-1* or *daf-16* dsRNA-producing plasmids in the presence (dashed lines) or absence (solid lines) of 1 mg/ml ethosuximide. Ethosuximide treatment significantly increased the lifespan of *dnj-14(ok237)* worms on vector control (*p* < 0.001), but had no significant effect on *daf-16* RNAi animals (*p* > 0.15) (n > 100 worms per strain/condition)

DEGs that showed at least a 2-fold change in all strains were considered the most significant ethosuximide-responsive genes and are listed in Table 1. Given the known neuroprotective effect of inhibiting the DAF-2 insulin/IGF signalling (IIS) pathway, it was notable that the upregulated genes included four members of the *dod* (downstream of *daf-16*) gene class associated with the DAF-2 signalling pathway; *asm-3*, a regulator of the DAF-2 pathway; and *ttr-44*, which is upregulated in long-lived *daf-2* mutants. The most strongly down-regulated transcripts were either uncharacterised genes or individual genes associated with diverse cellular processes, for example ubiquitination (*fbxb-66*). Seven of the up-regulated genes identified in our microarray experiments were chosen for validation using qRT-PCR. Significantly increased expression by qRT-PCR was observed for all 7 genes, thus confirming the microarray data (Fig. 3a). In contrast, qRT-PCR analysis showed no change in the expression level of *pph-6* (chosen as a negative control based on our microarray data) or of two normalising genes: *pmp-3* and *act-1*.

**Table 1:** Gene expression changes induced by ethosuximide in all microarray experiments

Selected criteria for inclusion were a FDR corrected *p*-value less than 0.01 and a calculated mean fold-change (FC) in expression greater than 2 or less than -2 in all four strains analysed. Red shading indicates up-regulated genes; green shading indicates down-regulated genes

Functional annotation enrichment analysis of DEGs shared by at least 3 treated strains was used to subdivide the ethosuximide-responsive transcripts into groups based on Gene Ontology (GO) identifiers (Additional file 6: Figure S4). Both DAVID and modMine GO analyses yielded a significant enrichment for GO terms related to “lipid glycosylation”, “lipid modification”, “oxidation reduction”, “determination of adult lifespan” and “chromatin assembly”. DEGs up-regulated in response to ethosuximide exposure clustered into four groups with significant enrichment scores (Additional file 6: Figure S4A). Cluster 1 contained eight cytochrome P450 genes (*cyp-14A3, cyp-34A2, cyp-34A9/dod-16, cyp-35B1/dod-13, cyp-35C1, cyp-33A1, cyp-35A2, cyp-35A3*), three short chain dehydrogenase genes (*dhs-23, dhs-26, dhs-2*) and an aldehyde dehydrogenase (*alh-5*). These enriched genes (Additional file 7: Figure S5) share annotation terms relating to oxidoreductase activity, ion binding and multicellular organismal ageing. Clusters 2 and 3 are overlapping and contain the same group of 6 putative UDP-glucuronosyl/glucosyl transferases (UGTs) involved in lipid and phase II metabolism (*F08A8.2, ugt-51, ugt-8, ugt-41, ugt-14, ugt-25*). Cluster 4 is associated with the term ageing and lifespan determination and comprised *cyp-34A9, cyp-35B1, dod-6, dod-3,* and *ftn-1*, which are amongst the most responsive downstream targets of DAF-16/FOXO; and *thn-1* and *spp.-1*. DEGs down-regulated in response to ethosuximide showed a significant enrichment to chromatin remodelling and related functional categories encompassing “cellular macromolecular complex assembly, chromatin assembly or disassembly, DNA packaging, nucleosome assembly, chromatin organisation, and chromatin assembly” (Additional file 6: Figure S4). Enriched genes (Additional file 7: Figure S5) include an H2B histone (*his-8*) and H1 linker histone variants (*his-24, hil-2, hil-3, hil-7*) which play roles in heterochromatin packaging and gene regulation. modMine publication enrichment analysis (Additional file 6: Figure S4C) further revealed a significant enrichment for DAF-16 target genes.

Analysis of the upstream promoter regions of ethosuximide-responsive DEGs using Regulatory Sequence Analysis Tools (RSAT) revealed that the most significantly enriched motif was CTTATCA (Fig. 3b). This is the consensus sequence for the DAF-16-associated element (DAE), which is overrepresented in the promoters of DAF-16-regulated target genes downstream of DAF-2 in the IIS pathway [34]. RSAT also identified accessory motifs that co-occurred with DAE, which appear to be core promoter sequence elements in *C. elegans* (Additional file 8: Figure S6). No additional motifs were identified using 3 other tools (MEME-DREME, SCOPE, BioProspector) and indeed DAE and its variants was the only regulatory sequence identified by all four sequence analysis tools (Additional file 8: Figure S6).

### Ethosuximide’s protective effect requires DAF-16

Subjecting *C. elegans* to mild stress can increase longevity via hormesis. However, comparing our microarray data with the literature (Additional file 9: Figure S7), it is evident that the transcriptomic effect of ethosuximide was inconsistent with a general stress response. Furthermore, ethosuximide did not induce oxidative stress and the consequent transcriptional induction of SKN-1 target genes, as evidenced by microarray data (Additional file 9: Figure S7), confirmatory qRT-PCR (Additional file 10: Figure S8A) and the lack of activation of a GFP reporter of the SKN-1 target, *gst-4* (Additional file 10: Figure S8B). Finally, paraquat and juglone applied at concentrations that have previously been shown to result in hormesis-induced lifespan extension did not rescue the *dnj-14* mutant despite inducing strong *Pgst-4*::GFP expression (Additional file 10: Figure S8C).

As our microarray data had revealed DAF-16 target gene modulation as a major consequence of ethosuximide application, we set out to test if DAF-16 was required for ethosuximide-mediated protection. We were unable to obtain stable lines of homozygous double mutants for both *dnj-14(ok237)* and *daf-16(mu86)*, as the putative double mutants exhibited severe developmental problems and extremely low brood size (Additional file 11: Figure S9), suggesting a synthetic lethal genetic interaction. We therefore used RNAi to knockdown expression of *daf-16*, which resulted in a significant reduction in lifespan of both wild type and *dnj-14(ok237)* worms (Fig. 3c; Additional file 12: Figure S10). Ethosuximide treatment of *dnj-14(ok237)* worms on L4440 vector control RNAi bacteria caused a robust lifespan extension; but, strikingly, *daf-16* RNAi abolished this effect (Fig. 3c). RNAi of *hsp-1*, which encodes an Hsp70 protein, also reduced lifespan, but this was significantly increased by ethosuximide (Additional file 12: Figure S10), indicating both that ethosuximide acts independently of *hsp-1* and that inhibition by *daf-16* RNAi is not a general consequence of RNAi. These data therefore suggest that DAF-16 is essential for the therapeutic action of ethosuximide.

To investigate if ethosuximide affects the subcellular localisation of DAF-16, we examined the effect of the drug on a strain containing a DAF-16-GFP reporter (Additional file 13: Figure S11). Although we could observe nuclear translocation of DAF-16-GFP in response to heat shock and starvation, we were unable to detect an obvious effect of ethosuximide. It is important to note, however, that this does not affect the conclusion that ethosuximide’s action requires DAF-16 activity. For example, the classical IIS mutant *age-1(hx546)*, which absolutely requires DAF-16 for lifespan extension, also does not exhibit increased nuclear DAF-16-GFP [35]. It is possible that the effect of ethosuximide on nuclear enrichment of DAF-16-GFP or the amount of nuclear DAF-16/FOXO required for the effects on lifespan might simply be below the detection threshold in this type of experiment.

### Ethosuximide modulates mammalian FOXO target gene expression

**Fig. 4:**
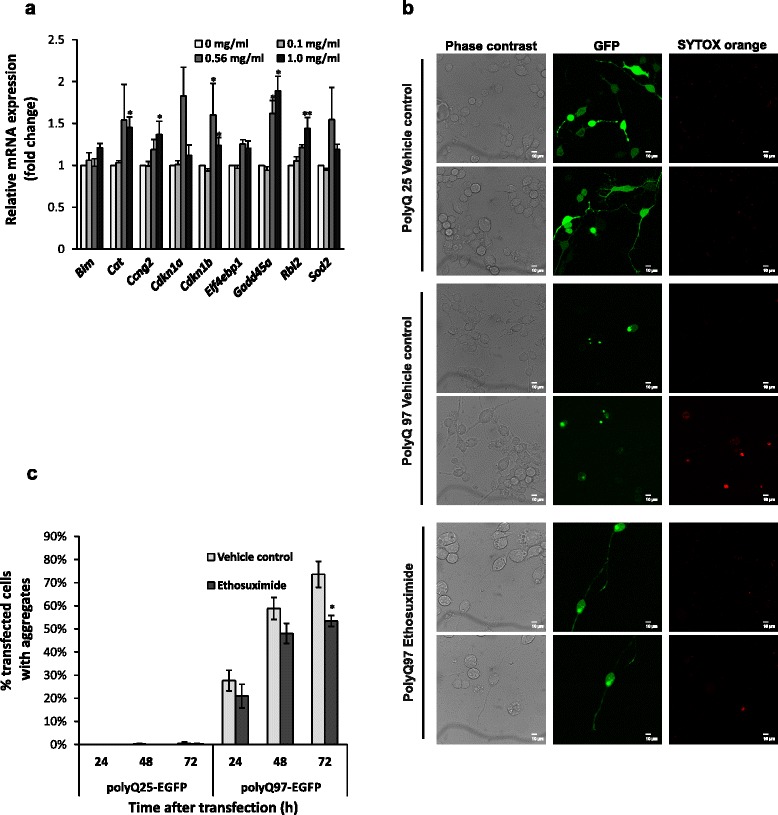
Ethosuximide induces FOXO target gene expression and reduces polyglutamine protein aggregation in mammalian neurons. **a** mRNA levels of classical FOXO target genes were analysed by qRT-PCR in differentiated mouse N2A neuroblastoma cells treated with the indicated concentrations of ethosuximide. Data are shown as mean ± SEM (n = 3; *p ≤ 0.05, **p ≤ 0.01). (**b**-**c**) Ethosuximide reduces polyglutamine protein aggregation. **b** Visualisation of EGFP-tagged non-pathological (polyQ25) and pathological (polyQ97) polyglutamine tracts in N2A cells 72 h post-transfection. Phase contrast, GFP (green) and SYTOX orange staining (red, to identify dead cells) confocal images are shown to illustrate that aggregates are specific to polyQ97 and that ethosuximide redistributed polyQ97-EGFP away from aggregates into the cytoplasm and neuronal processes C) Quantification of polyQ aggregation. The number of polyQ-EGFP transfected N2A cells bearing fluorescent aggregates as a percentage of the total number of viable transfected (green) cells was counted at the indicated post-transfection times. Cell/aggregate counting was performed manually and confirmed using ImageJ software if cells were sufficiently sparse to allow this. Data are shown as mean ± SEM (n = 3, counting ~100 cells in each experiment; *p < 0.05)

Given that DAF-16/FOXO is conserved between *C. elegans* and mammals, we tested whether ethosuximide could affect the transcriptional activity of mammalian DAF-16 homologues (FOXO1, FOXO3, and FOXO4). FOXOs have been shown to modulate cell cycle arrest, apoptosis, autophagy, angiogenesis, differentiation, stress resistance, insulin signalling, stem cell maintenance and metabolism [36]. Nine classical mammalian FOXO target genes involved in cell cycle regulation (*Ccng2, Cdkn1a, Cdkn1b, Rbl2*), DNA repair (*Gadd45a*), apoptosis (*Bim*), stress response (*Cat, Sod2*) and insulin signalling (*Eif4ebp1*) were selected and their mRNA levels in a differentiated mouse neuroblastoma cell line (N2A) following ethosuximide exposure were then measured using qRT-PCR. There was no significant change in gene expression at 0.1 mg/ml, but at 0.56 mg/ml (the optimal concentration that stimulates neuronal differentiation in stem cells [37]) and 1 mg/ml (the optimal concentration in our worm ND models) ethosuximide significantly up-regulated the mRNA expression of FOXO target genes involved in cell cycle regulation (*Ccng2, Cdkn1b, Rbl2*) and DNA damage repair (*Gadd45a*) (Fig. 4a). Therefore, the ability of ethosuximide to modulate DAF-16/FOXO target gene expression is conserved from worms to mammals.

### Ethosuximide suppresses protein aggregation in mammalian neurons

To determine if ethosuximide could also affect proteostasis in mammalian neurons, we transfected N2A cells with EGFP-tagged polyglutamine (polyQ) constructs and monitored aggregate formation over time in culture [38]. PolyQ25 barely aggregated either in the presence or absence of ethosuximide, as GFP staining was evenly distributed throughout the cytosol (Fig. 4b). In contrast, there was a progressive increase in the number of cells with polyQ97 aggregates, with intracellular GFP punctae readily observable in the cytoplasm of most polyQ97-transfected N2A cells after 72 h (Fig. 4b, Additional file 14: Figure S12). When aggregates appeared, cells tended to round up and GFP-fluorescence in the cytosol was restricted to bright punctae. Ethosuximide treatment appeared to antagonise aggregate formation and to increase diffuse cytosolic GFP staining in the soma and neuronal processes, suggesting that it enhanced the solubility of polyQ97. To quantify this, individual GFP aggregates were counted and the percentage of polyQ-EGFP transfected N2A cells bearing fluorescent aggregates was determined. Ethosuximide treatment significantly reduced the fraction of transfected cells containing aggregates by 20 % compared with the vehicle control (Fig. 4c), although neuronal viability as quantified by SYTOX Orange staining was unaffected by ethosuximide (Additional file 15: Figure S13). Therefore, ethosuximide can antagonise protein aggregation in worm neurons *in vivo* and in mammalian neurons *in vitro*.

## Discussion

Neurodegenerative diseases are increasingly common and exert large costs on society. However, no disease-modifying therapies for these devastating disorders are currently available. Hence, there is considerable current interest in the idea of repurposing existing medicines for the treatment of neurodegeneration [8]. Drugs that can mitigate the impact of common pathological processes such as protein misfolding/aggregation that underlie multiple neurodegenerative diseases could be especially useful therapeutics. Ethosuximide may be a candidate for such a repurposing approach. We have shown here that ethosuximide ameliorates the phenotypes of two distinct worm neurodegenerative disease models based on deletion of an endogenous neuroprotective gene (*dnj-14*) and pan-neuronal expression of a disease-associated mutant Tau protein. Furthermore, an independent study has recently reported a neuroprotective effect of ethosuximide against human mutant mTDP-43-mediated proteotoxicity in a transgenic *C. elegans* model of amyotrophic lateral sclerosis [15]. Importantly, we have discovered that ethosuximide also reduces polyglutamine protein aggregation in mammalian neuroblastoma cells and acts by modulating DAF-16/FOXO target gene expression in both worms and mammalian neurons. These evolutionarily conserved effects on so many different neurodegenerative disease models suggest that ethosuximide merits consideration for therapeutic applications in patients. Indeed, given the difficulty of distinguishing between tau- and TDP-43-associated frontotemporal dementia in the clinic, a drug with potential to protect against both pathologies could be particularly useful. Future experiments in mouse neurodegeneration models should therefore be given a high priority in order to validate ethosuximide’s repurposing potential for treatment of human neurodegenerative diseases.

The mechanism by which ethosuximide exerts its anti-epileptic action is unclear and controversial. Ethosuximide has long been thought to act by blocking T-type calcium channels; however, more recent work has suggested that actions on other voltage-sensitive ion channels, such as sodium and potassium channels, are involved in the therapeutic effect of ethosuximide [30]. Our finding that the beneficial effects of ethosuximide on motility and longevity in a frontotemporal dementia model persist in strains harbouring a null mutation in the *C. elegans* T-type calcium channel, *cca-1*, suggests that ethosuximide’s neuroprotective activity is not mediated by inhibition of T-type channels. This conclusion is supported by previous work in *C. elegans* showing that lifespan extension by ethosuximide is unaffected by a different mutant allele of *cca-1* [39]. Although we cannot rule out redundancy with other channel subunit homologs that share a putative ethosuximide binding site, given that the *C. elegans* genome does not encode voltage-sensitive sodium channels [40], it seems likely that ethosuximide ameliorates neurodegeneration via a distinct mechanism.

Ethosuximide has previously been suggested to extend lifespan in wild type worms via inhibition of chemosensory neurons [39, 41], resulting in a perceived state of dietary restriction and hence lifespan increase [39]. However, ethosuximide’s ability to extend lifespan persists even under maximal dietary restriction conditions [42], thus arguing against this mechanism. In our study, lifespan in wild type control worms was unaffected by ethosuximide, but nevertheless the protective effect of ethosuximide in worm neurodegeneration models is unlikely to involve inhibition of chemosensory neurons, as the *dnj-14* model used in this study already exhibits profound chemosensory impairment, which is actually rescued by ethosuximide.

The transcriptomic analysis reported here suggests instead that ethosuximide acts by modulating the expression of DAF-16/FOXO target genes. DAF-16/FOXO is a conserved transcription factor acting downstream of DAF-2 in the IIS pathway [36]. Reducing IIS pathway activity by inhibiting DAF-2 causes an increase in DAF-16 activity and the consequent modulation of DAF-16 target gene expression leads to phenotypic changes such as increased lifespan. In addition to its pivotal role in longevity and stress resistance, reduced IIS pathway activity has been shown to confer neuroprotection in nematode neurodegeneration models based on expression of Aβ [43], TDP-43 [44] and polyQ [45]; and also in mouse Aβ models [46]. Our finding that the neuroprotective effect of ethosuximide *in vivo* was dependent on DAF-16 function is consistent with these studies and also with our transcriptomic analysis. It is also consistent with the recent observation that ethosuximide’s protective effect on a *C. elegans* amyotrophic lateral sclerosis model requires DAF-16 [15], but contrasts with earlier work showing that ethosuximide still increases lifespan in *daf-16* mutants [41]. Although we observed little effect of ethosuximide on *skn-1* pathway targets or on oxidative stress detoxification genes such as catalase, superoxide dismutases and peroxiredoxins, genes that are commonly regulated by DAF-16 and oxidative stress showed some enrichment (Additional file 9: Figure S7), which may be relevant given the established links between oxidative stress and protein aggregation in neurodegenerative disease models. Ethosuximide may mimic some of the effects of reduced IIS by inducing the expression of neuroprotective DAF-16 target genes, in particular those containing the DAE motif [47].

Analogous to its ability to regulate DAF-16 target gene expression and modulate protein aggregation in *C. elegans*, we found that ethosuximide induces transcription of FOXO target genes and confers protection against polyQ aggregation in mammalian neuronal cell lines. This therefore suggests an evolutionary conservation of DAF-16/FOXO-regulated ethosuximide responses. The DAF-16 homologue FOXO3a is highly expressed in adult brain, and plays key regulatory roles in neuronal survival under basal, stress and disease conditions [48, 49]. Furthermore, FOXO3a contributes to neuroprotection by Sirt1 in a striatal cell model of Huntington’s disease [50] and moderate FOXO3 activation was recently shown to oppose α-synuclein accumulation and proteotoxicity [51].

Ethosuximide is cheap to manufacture, has been widely prescribed for several decades, is well tolerated at high doses and has a good safety record – indeed, it is often used in children to treat absence seizures [52]. Furthermore, it has >95 % bioavailability and freely crosses the blood-brain barrier, with levels of the drug in cerebrospinal fluid and multiple brain regions being similar to those found in plasma [52]. Optimal improvement in the *C. elegans* models described here was seen at an externally applied concentration of 1-2 mg/ml, which equates to a measured internal concentration within the worm of 15-30 μg/ml [41]; while effects in mammalian neurons were seen at 0.5-1 mg/ml. These values are close to the therapeutic dose range of ethosuximide for epilepsy in humans (40-100 μg/ml) [52], suggesting that neuroprotective doses could be achieved in patients. Ethosuximide may therefore be a candidate drug for repurposing as a treatment for neurodegenerative diseases.

## Conclusion

We have shown here that ethosuximide has neuroprotective activity on multiple neurodegeneration models ranging from worms to mammalian cultured neurons. Furthermore, we have revealed that this anti-epileptic drug has an evolutionarily conserved effect in modulating DAF-16/FOXO target gene expression; and that DAF-16 is essential for ethosuximide’s therapeutic action in the worm dnj-14 model. Ethosuximide is off-patent, has a good safety record and freely permeates the blood-brain barrier; and so could potentially be rapidly repurposed for other neurological indications. Future experiments in established mouse neurodegeneration models should therefore be a high priority to validate the repurposing potential of this well-established anti-epileptic drug for treatment of human neurodegenerative diseases.

## Materials and methods

### Experimental design

This study aimed to investigate if ethosuximide had general neuroprotective activity and to shed light on its mechanism of action. To address this question, we initially performed drug screening in the *dnj-14* worm neurodegeneration model that we developed, using chemotaxis and lifespan assays. Subsequently, the effect of ethosuximide was investigated on a transgenic worm model of FTD, using motility and lifespan assays and determination of tau protein aggregation by western blot analyses. Transcriptomic effects of ethosuximide were analysed on identical numbers of age-matched control and treated animals using Affymetrix *C. elegans* GeneChips, then validated by qRT-PCR using independent biological samples. Differentially expressed genes were identified using the statistical analysis tools R, Affy and limma. Functional annotation and clustering of putative ethosuximide target genes was performed using the DAVID and modENCODE Modmine bioinformatic suites; and predictions tested by *C. elegans* RNAi experiments. Evolutionary conservation of ethosuximide’s actions was assessed by qRT-PCR analysis and fluorescence microscopy imaging of GFP-tagged polyglutamine aggregation using retinoic acid-differentiated mouse neuroblastoma cells.

### Maintenance and propagation of *C. elegans* strains

*C. elegans* strains were cultured on nematode growth medium (NGM) culture plates seeded with the *E. coli* strain OP50 at 20 °C unless stated otherwise. *C. elegans* strains used in this study are detailed in Additional file 16: Table S2, and were either obtained from Caenorhabditis Genetics Center (CGC, University of Minnesota, USA, generously provided by the indicated labs that generated them, or constructed in-house.

#### Drug treatment

All chemicals were obtained from Sigma Chemical Co. (St. Louis, MO). Ethosuximide was prepared as a concentrated stock solution in phosphate buffered saline (PBS) and was added to molten NGM before pouring into petri dishes. Vehicle control NGM plates containing equivalent amounts of PBS were prepared at the same time. Freshly poured drug plates were stored in the dark at 4 °C until 1-2 days before use and then moved to room temperature and seeded with *E. coli* OP50. Worms were grown for two generations on drug plates to ensure effective exposure. 10-20 gravid adults of test strains were cultured in the presence of the drug to lay eggs for 6 h and then removed to set the eggs in synchrony. Plates were grown at 20 °C for three days and selected for analysis at the L4 stage (defined as day 0).

#### Behavioural and lifespan assays

Motility and chemotaxis assays were performed as previously described [18, 28] on developmentally synchronised worms. For lifespan assays, animals were scored every 1-2 days for survival by examining for touch provoked movement. Worms which did not display spontaneous movement or response to repeated touching were scored as dead. Worms were transferred onto fresh plates every 2 -4 days until the cessation of progeny production. Dead worms that displayed internally hatched progeny, an extruded gonad, or desiccation caused by crawling off the agar were excluded from the data.

#### Sequential extraction of Tau protein

A detailed description is provided in Additional file 17. Vehicle- and ethosuximide-treated Tau V337M worms were lysed and separated into soluble and insoluble (RIPA detergent-extractable) fractions, using previously described methods [29]. Fractions were separated by SDS-PAGE and western blotted using anti-human Tau T46 (Invitrogen) and anti-actin (Sigma) antibodies. The abundance of Tau protein in each fraction was quantified by densitometry and normalised against beta-actin. Total Tau levels in lysates were expressed as the percentage of actin-normalised Tau relative to vehicle control lysates; Tau levels in sequentially extracted fractions were expressed as the percentage of actin-normalised Tau relative to the sum of both fractions (soluble + RIPA) combined.

### Genetic crosses

N2 males were mated with CK10 P_*aex-3*_::Tau V337M; P_*myo-2*_*::GFP* hermaphrodites; and the resulting transgenic male offspring were then back-crossed to isolate homozygous GFP (-Tau)-expressing males. These were then mated with *cca-1(ad1650)* hermaphrodites and their progeny genotyped to identify homozygous transgenic double mutants via PCR using primers flanking the *cca-1(ad1650)* deletion [forward: 5’-CCGCAATTTGCCCTCCACAT-3’; reverse: 5’-ATGAGGATGGCGAAGAGGACC-3’] and primers based on the human *MAPT* gene that encodes Tau (forward: 5’-CAAGCTCGCATGGTCAGT AA-3’; reverse: 5’-TTCTCAGTGGAGCCGATC TT-3’).

### RNA interference

RNAi experiments were conducted using a previously described feeding protocol [53]. Worms were grown on HT115 (DE3) bacteria expressing pG-L4440-based plasmids from the *C. elegans* Open Reading Frame (ORF) RNAi feeding library (Source Bioscience, Nottingham, UK). ORF inserts were verified by restriction endonuclease digest prior to use. RNAi treatments were performed as whole-life treatments and second generation RNAi-fed worms were synchronised on fresh RNAi plates supplemented with test compound (ethosuximide) or vehicle (PBS). Feeding of HT115 carrying the pG-L4440 vector without an insert (L4440) served as the empty vector negative control.

### Transcriptomic work

A detailed description is provided in Additional file 17. Briefly, worms were grown on vehicle- or ethosuximide-containing NGM plates until age day 6. RNA was then extracted, amplified, labelled and hybridised to Affymetrix *C. elegans* GeneChips (Affymetrix, High Wycombe, UK). Three independent biological replicate samples were used for each strain and condition (24 gene arrays in total). Sequences 200 bp upstream of *C. elegans* DEGs were analysed to identify shared DNA motifs using Regulatory Sequence Analysis Tools (RSAT; http://rsat.bigre.ulb.ac.be/rsat/), MEME (http://meme-suite.org/), BioProspector (http://seqmotifs.stanford.edu) and SCOPE (http://genie.dartmouth.edu/scope/). Microarray data are available in the ArrayExpress database (www.ebi.ac.uk/arrayexpress) under accession number E-MTAB-3919.

### Quantitative real-time PCR (qRT-PCR)

A detailed description is provided in Additional file 17 and all qRT-PCR primers are listed in Additional file 18: Table S3. *act-1* and *pmp-3,* which showed no significant changes in microarray analysis and have been extensively used as normalising genes in differential expression studies in *C. elegans* [54], were chosen as reference genes. RNA was extracted and used to prepare cDNA, which was then used for SYBR® Green-based real-time PCR using an IQ5 detection system (Bio-Rad). All qRT-PCR reactions were carried out on 3 biological replicates with three technical replications. Transcript expression was analysed using Bio-Rad CFX Manager 3.0 software and relative expression was calculated by normalising the Ct values for test genes to reference genes.

### Mammalian cell methods

A detailed description is provided in Additional file 17. For qRT-PCR analysis, mouse Neuro2A (N2A) neuroblastoma cells were cultured on 6-well plates and treated with retinoic acid to induce neuronal differentiation. After 24 h, N2A cells were treated with vehicle control (PBS) or increasing concentrations of ethosuximide for 5 h. Total RNA was then isolated, DNase-treated and reverse transcribed to cDNA. qRT-PCR was run as described above normalising to the reference genes glyceraldehyde-3-phosphate dehydrogenase (GAPDH) and β-actin (ACTB). Three independent biological replicates were used for qRT-PCR. For microscopy, N2A cells were grown on glass-bottom 35-mm dishes and treated with retinoic acid to stimulate neuronal differentiation prior to transient transfection with polyQ97-eGFP or polyQ25-eGFP plasmids construct using PromoFectin transfection reagent (PromoKine). Live transfected cells were imaged using a Leica AOBS SP2 microscope (Leica Microsystems, Heidelberg, Germany) and the percentage of live cells with visible GFP aggregates determined at 24, 48 and 72 h post-transfection. A minimum of 50 cells per condition were counted manually and the calculated percentage of transfected cells containing GFP aggregates was derived from three independent experiments. Neuronal viability was visualised by SYTOX Orange® staining (Invitrogen) and the percentage cell death calculated by manually counting the number of labelled cells from a total of at least 800 cells per condition imaged over three independent experiments.

### Statistical analysis

Statistical analysis of lifespan data was performed using the log-rank (Mantel-Cox) test, and mortality at specific time points was compared using Fisher’s exact test. For behavioural assays, if two data sets were being directly compared, Student’s *t*-test was used; for comparison of multiple data sets, one-way analysis of variance (ANOVA) was used. For transcriptomic data, a significance test of the estimated fold change for each contrast was performed by the empirical Bayes function packed in limma, and *p*-values were adjusted using the Benjamini and Hochberg false discovery rate (FDR) control approach to deal with the effect of multiple tests [55]. Genes with a FDR corrected *p*-value equal to or less than 0.01 and fold change greater than 2 were deemed significantly differentially expressed.

## Additional files

Additional file 1: Table S1. Statistical analysis of lifespan experiments in *C. elegans*. (PDF 103 kb) Additional file 2: Figure S1. Ethosuximide does not increase lifespan or sensorimotor function in wild type *C. elegans* strains. (PDF 1430 kb) Additional file 3: Figure S2. Transcriptomics workflow. (PDF 1368 kb) Additional file 4: Figure S3. Whole-genome expression profiling of *dnj-14(ok237)*, *dnj-14(tm3223)*, CZ1200 and N2 strains treated with ethosuximide. (PDF 1542 kb) Additional file 5: 
**Dataset S1.**Transcriptomic data. (XLSX 939 kb) Additional file 6: Figure S4. Functional enrichment analysis of common DEGs. (PDF 1365 kb) Additional file 7: Figure S5. DEGs derived from highly enriched functional clusters. (PDF 33 kb) Additional file 8: Figure S6. Ethosuximide-responsive genes are enriched for a DAF-16 Associated Element (DAE) motif. (PDF 145 kb) Additional file 9: Figure S7. Data set associations. (PDF 1374 kb) Additional file 10: Figure S8. Ethosuximide does not induce general stress and hormesis. (PDF 1626 kb) Additional file 11: Figure S9. Negative genetic interactions between *dnj-14* and *daf-16*. (PDF 1580 kb) Additional file 12: Figure S10. Ethosuximide action is independent of *hsp-1*. (PDF 98 kb) Additional file 13: Figure S11. Ethosuximide does not cause obvious nuclear translocation of GFP-tagged DAF-16. (PDF 1626 kb) Additional file 14: Figure S12. Ethosuximide reduces polyglutamine protein aggregation in mammalian neurons. (PDF 2095 kb) Additional file 15: Figure S13. Ethosuximide has no significant effect on cytotoxicity in mammalian neurons. (PDF 33 kb) Additional file 16: Table S2.
*C. elegans* strains used in this study. (PDF 12 kb) Additional file 17:
**Supplementary Methods.** (PDF 70 kb) Additional file 18: Table S3. Sequences of oligonucleotides used in this study. (PDF 27 kb)
