# Erratum to: Ethosuximide ameliorates neurodegenerative disease phenotypes by modulating DAF-16/FOXO target gene expression

**DOI:** 10.1186/s13024-015-0051-6

**Published:** 2015-10-23

**Authors:** Xi Chen, Hannah V. McCue, Shi Quan Wong, Sudhanva S. Kashyap, Brian C. Kraemer, Jeff W. Barclay, Robert D. Burgoyne, Alan Morgan

**Affiliations:** Department of Cellular and Molecular Physiology, Institute of Translational Medicine, University of Liverpool, Crown St, Liverpool, L69 3BX UK; Geriatrics Research Education and Clinical Center, Seattle Veterans Affairs Puget Sound Health Care System and University of Washington Department of Medicine, 1660 South Columbian Way, Seattle, 98108 WA USA; Present Address: Centre for Neurodegenerative Science, Van Andel Research Institute, 333 Bostwick Avenue NE, Grand Rapids, 49503 MI USA

The original version of this article [1] unfortunately contained a mistake. The author list contained a spelling error for the author Hannah V. McCue. The original article has been corrected for this error. The corrected author list is given below:

Xi Chen, Hannah V. McCue, Shi Quan Wong, Sudhanva S. Kashyap, Brian C. Kraemer, Jeff W. Barclay, Robert D. Burgoyne and Alan Morgan
