# Supplementary material for: Ethosuximide ameliorates neurodegenerative disease phenotypes by modulating DAF-16/FOXO target gene expression
Source: Mol Neurodegener. 2015 Sep 29;10:51. doi: 10.1186/s13024-015-0046-3 (PMC4587861; doi:10.1186/s13024-015-0046-3)
Supplement: Additional file 1: Table S1. — Statistical analysis of lifespan experiments in C. elegans. (PDF 103 kb) [file 13024_2015_46_MOESM1_ESM.pdf]

Strains	Drug treatments$^{\Delta}$ (mg/ml)	Mean lifespan ± S.E.M (days)	% Change in mean lifespan§	*p*-value	75% mortality (days)	90% mortality (days)	Maximum (days)	Total number of worms analysed
N2	PBS control	19.69 ± 1.05	-	-	27	29	31	53
	Juglone (40 μM)	16.43 ± 0.55**	-16.56%	0.0021	18†††	23†	25	52
	Succinimide (1)	15.91 ± 0.57**	-19.20%	0.0003	18†††	21†	25	52
	Ethosuximide (1)	20.39 ± 1.02	3.56%	0.3966	27	29	33	54
	PBS control	18.55 ± 0.64	-	-	22	24	25	52
	Ethosuximide (2)	18.79 ± 1.06	1.29%	0.5061	24	25	29	52
	Ethosuximide (4)	17.12 ± 0.98	-7.71%	0.5000	21	23	26	52
N2; *L4440* (RNAi)	PBS control	19.48 ± 0.30	-	-	22	25	27	131
	Ethosuximide (1)	19.08 ± 0.38	-2.05%	0.9164	22	25	27	130
N2; *hsp-1* (RNAi)	PBS control	12.19 ± 0.24***	-	0.0e+00	14†††	15	17	116
	Ethosuximide (1)	14.35 ± 0.38***	17.72%	2.2e-08	17†††	20	25	118
N2; *daf-16a* (RNAi)	PBS control	15.73 ± 0.33***	-	0.0e+00	18†††	20††	22	128
	Ethosuximide (1)	15.90 ± 0.33	1.08%	0.8868	18	20	25	128
dnj-14(ok237)	PBS control	15.87 ± 0.62	-	-	18	21	23	56
	Juglone (40 μM)	18.01 ± 0.57*	13.48%	0.0070	21	23	25	58
	Succinimide (1)	16.71 ± 0.51	5.29%	0.3586	18	21	25	54
	Ethosuximide (1)	21.56 ± 0.79***	35.85%	1.1e-07	21	23	25	58
	PBS control	15.76 ± 0.61	-	-	17	21	27	52
	Ethosuximide (0.0001)	16.66 ± 0.76	5.71%	0.4174	19†	23†	24	52
	Ethosuximide (0.001)	18.40 ± 0.84*	16.75%	0.0217	21††	24††	26	52
	Ethosuximide (0.01)	16.59 ± 0.60	5.27%	0.3757	19	22	25	50
	Ethosuximide (0.1)	17.46 ± 0.78	10.79%	0.1722	21	25†	26	53
	Ethosuximide (0.2)	19.27 ± 0.78**	22.27%	0.0017	23†	24	29	52
	Ethosuximide (0.5)	20.15 ± 0.98**	27.86%	0.0005	24††	26††	32	52
	Ethosuximide (1)	22.26 ± 1.25***	41.24%	2.6e-06	29††	32†	34	112
	Ethosuximide (2)	21.11 ± 1.21**	33.95%	0.0002	26††	32†	33	52
	Ethosuximide (4)	18.73 ± 0.75**	18.85%	0.0038	22††	24	26	55
dnj-14(ok237); *L4440* (RNAi)	PBS control	16.81 ± 0.32***	-	3.5e-09	20†††	22†	25	118
	Ethosuximide (1)	21.23 ± 0.44***	26.29%	1.2e-06	25††	27†	31	125
dnj-14(ok237); *hsp-1* (RNAi)	PBS control	14.16 ± 0.28***	-	0.0e+00	15†††	17††	22	120
	Ethosuximide (1)	17.39 ± 0.39***	22.81%	0.0e+00	20†††	25†††	27	119
dnj-14(ok237); *daf-16a* (RNAi)	PBS control	15.37 ± 0.37*	-	0.0113	18	20	22	114
	Ethosuximide (1)	16.23 ± 0.33	5.60%	0.1518	18	20	22	126

Strains	Drug treatments[Δ] (mg/ml)	Mean lifespan ± S.E.M (days)	% Change in mean lifespan[§]	*p*-value	75% mortality (days)	90% mortality (days)	Maximum (days)	Total number of worms analysed
lin-25(n756)X; *juIs76*[P_{unc-25}::GFP + *lin-15(+)*]	PBS control	15.28 ± 0.41	-	-	18	20	22	102
	Ethosuximide (0.1)	16.02 ± 0.59	4.84%	0.1813	19	20	22	50
	Ethosuximide (0.2)	14.53 ± 0.58	-4.91%	0.5148	18	20	22	50
	Ethosuximide (0.5)	14.99 ± 0.79	-1.90%	0.8618	20	21	22	50
	Ethosuximide (1)	14.61 ± 0.65	-4.38%	0.8320	18	20	22	52
	Ethosuximide (2)	14.56 ± 0.76	-4.71%	0.5504	18†	19†	22	52
bkIs10 [P_{aex-3}::*h4R1N TauV337M* + P_{myo-2}::*GFP*]	PBS control	10.94 ± 0.27	-	-	13	14	16	102
	Ethosuximide (0.0001)	13.18 ± 0.49**	20.48%	0.0002	16	17	20	52
	Ethosuximide (0.001)	13.51± 0.27***	23.49%	1.1e-06	15†	16	17	52
	Ethosuximide (0.01)	13.80 ± 0.52***	26.14%	5.6e-06	15†	20	21	52
	Ethosuximide (0.1)	13.79 ± 0.36***	26.05%	8.8e-09	16†††	18†	20	102
	Ethosuximide (0.2)	14.04 ± 0.36***	28.34%	2.4e-09	16†††	17†	20	102
	Ethosuximide (0.5)	13.76 ± 0.34***	25.78%	5.6e-09	15†††	16†	19	102
	Ethosuximide (1)	14.17 ± 0.37***	29.52%	0.0e+00	16††	19	20	102
	Ethosuximide (2)	15.50 ± 0.40***	41.68%	0.0e+00	18†††	20†	21	102
	PBS control	11.28 ± 0.30	-	-	13	14	17	92
	PBS control + Kanamycin	15.50 ± 0.46***	37.41%	0.0e+00	17†††	19†	25	81
	Ethosuximide (1)	16.71 ± 0.51***	48.14%	0.0e+00	21	23	25	80
	Ethosuximide (1) + Kanamycin	19.41 ± 0.60***	72.07%	0.0e+00	23	25	29	80
cca-1(ad1650)	PBS control	17.50 ± 0.55	-	-	21	24	27	108
	Ethosuximide (1)	17.82 ± 0.57	1.83%	0.5423	21	26	27	107
bkIs10 [P_{aex-3}::*h4R1N TauV337M* + P_{myo-2}::*GFP*]	PBS control	11.45 ± 0.33	-	-	14	15	17	116
	Ethosuximide (1)	14.80 ± 0.43***	29.26%	6.5e-09	17††	19†	22	116
CK10; *cca-1(ad1650)*	PBS control	11.27 ± 0.31	-	-	14	15	19	114
	Ethosuximide (1)	14.27 ± 0.35***	26.62%	2.2e-09	16	17††	23	112

Table S1: Statistical analysis of lifespan extension in *C. elegans*. Adult mean lifespan ± SEM in days observed in lifespan analysis. The strains and concentrations of the drugs tested were indicated. Lifespan experiments were carried out at 20°C. Online Application for the Survival Analysis of Lifespan Assays (OASIS; http://sbi.postech.ac.kr/oasis/introduction/) (Yang *et al.*, 2011) was used for statistical analysis and to determine means and percentiles. Mean lifespans were compared using the log-rank (Mantel-Cox) test (*p<0.05; **p<0.005;***p<0.0001), and mortality at more specific time points was compared using Fisher's exact test ($^{†}p$<0.05 $^{††}p$<0.005; $^{†††}p$<0.0001). ∆Drug treatments were from conception until death. PBS was used to solubilise ethosuximide, and PBS alone was the control treatment for ethosuximide. For the antibiotic kanamycin treatment of OP50 *E.coli*, 80 µl of 10 mM kanamycin solubilised by water was added after 24 hours of bacterial growth as described, kanamycin alone was the control treatment for kanamycin + ethosuximide combined treatment. §The percentage change and the statistical significance of this change were determined by comparing the experimental ethosuximide treatments to PBS control. The experimental treatment kanamycin + ethosuximide was compared to kanamycin.
