# Supplementary material for: Ethosuximide ameliorates neurodegenerative disease phenotypes by modulating DAF-16/FOXO target gene expression
Source: Mol Neurodegener. 2015 Sep 29;10:51. doi: 10.1186/s13024-015-0046-3 (PMC4587861; doi:10.1186/s13024-015-0046-3)
Supplement: Additional file 2: Figure S1. — Ethosuximide does not increase lifespan or sensorimotor function in wild type C. elegans strains. (PDF 1430 kb) [file 13024_2015_46_MOESM2_ESM.pdf]

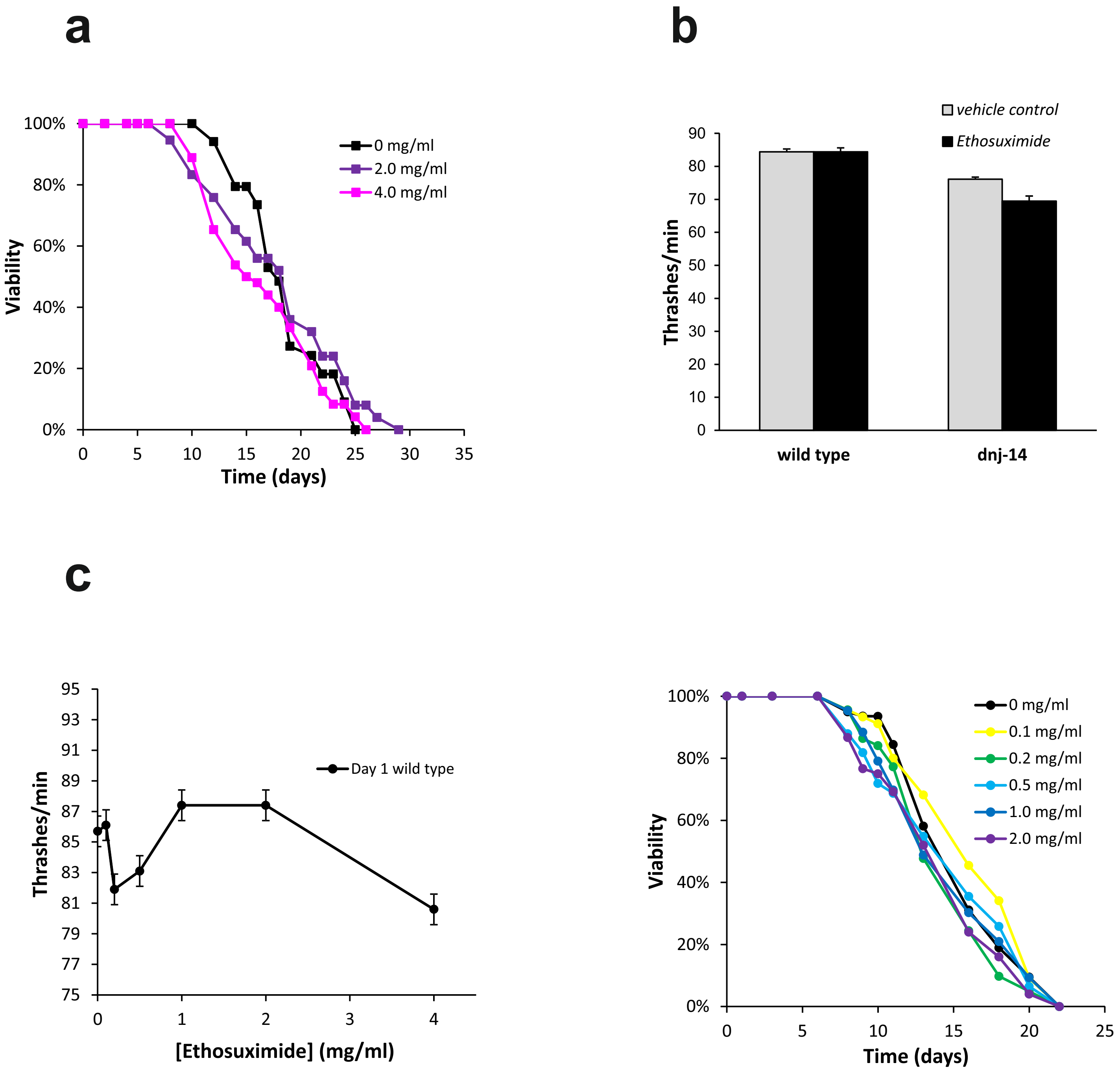

[illegible] ***C. elegans* strains.** (A) Viability of age-synchronised Bristol N2 wild type control animals grown in the presence of the indicated concentrations of ethosuximide was determined (n = 50-55 worms for each concentration). (B, C) Thrashing in solution was measured in the wild type strain CZ1200 grown in the presence of varying concentrations of ethosuximide (B) and in wild type N2 and *dnj-14(tm3223)* worms grown in the presence of 1 mg/ml ethosuximide (C). Data are shown as mean ± SEM (n = 20-30 worms per concentration). (D) Viability of age-synchronised CZ1200 control animals grown in the presence of the indicated concentrations of ethosuximide was determined (n = 50-102 worms for each drug concentration).
