# Supplementary figures and images for: Ethosuximide ameliorates neurodegenerative disease phenotypes by modulating DAF-16/FOXO target gene expression
Source: Mol Neurodegener. 2015 Sep 29;10:51. doi: 10.1186/s13024-015-0046-3 (PMC4587861; doi:10.1186/s13024-015-0046-3)

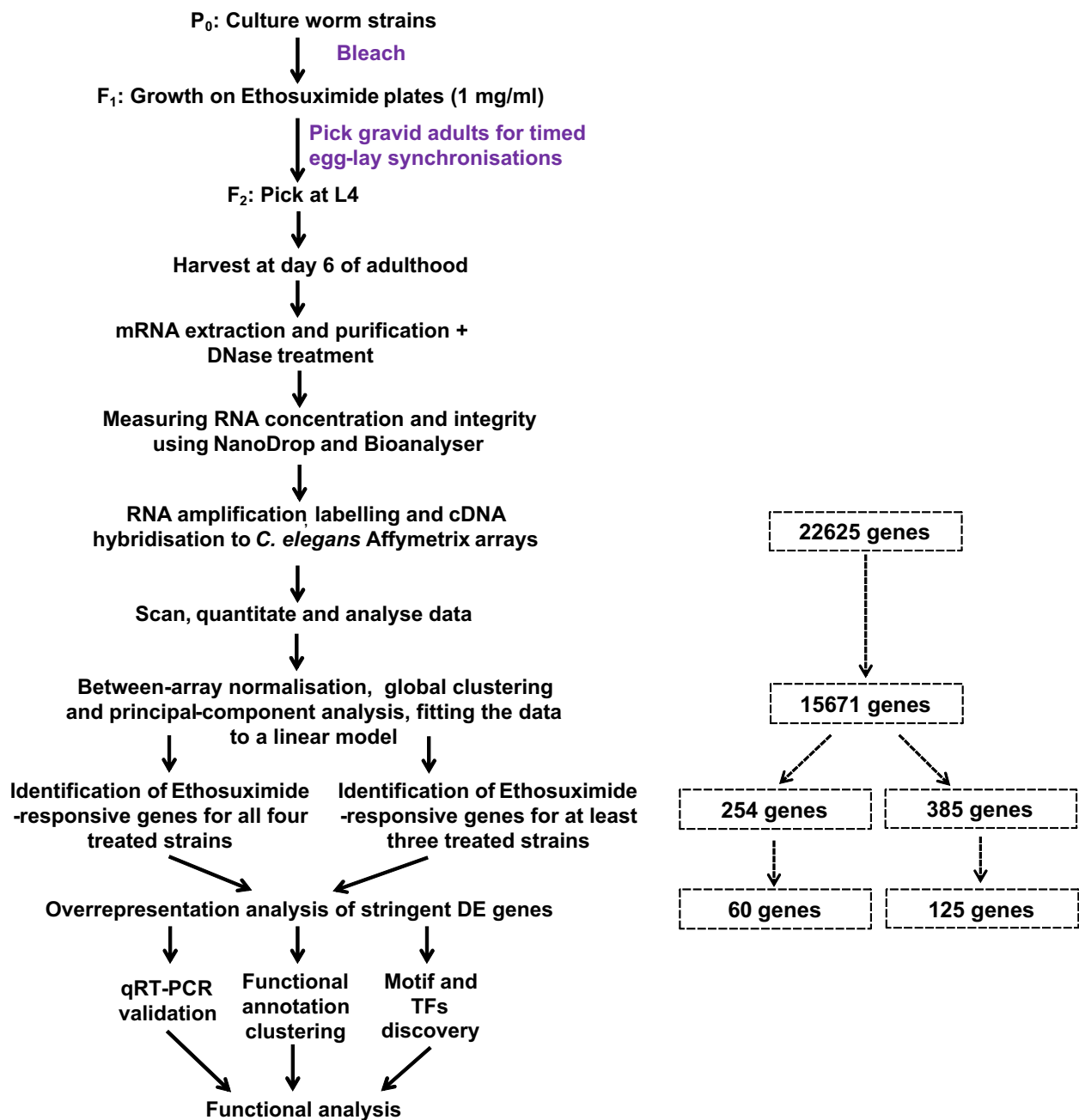

Figure S2. Transcriptomics workflow.

Supplement: Additional file 3: Figure S2. — Transcriptomics workflow. (PDF 1368 kb) [file 13024_2015_46_MOESM3_ESM.pdf]
