# Supplementary material for: Ethosuximide ameliorates neurodegenerative disease phenotypes by modulating DAF-16/FOXO target gene expression
Source: Mol Neurodegener. 2015 Sep 29;10:51. doi: 10.1186/s13024-015-0046-3 (PMC4587861; doi:10.1186/s13024-015-0046-3)
Supplement: Additional file 4: Figure S3. — Whole-genome expression profiling of dnj-14(ok237), dnj-14(tm3223), CZ1200 and N2 strains treated with ethosuximide. (PDF 1542 kb) [file 13024_2015_46_MOESM4_ESM.pdf]

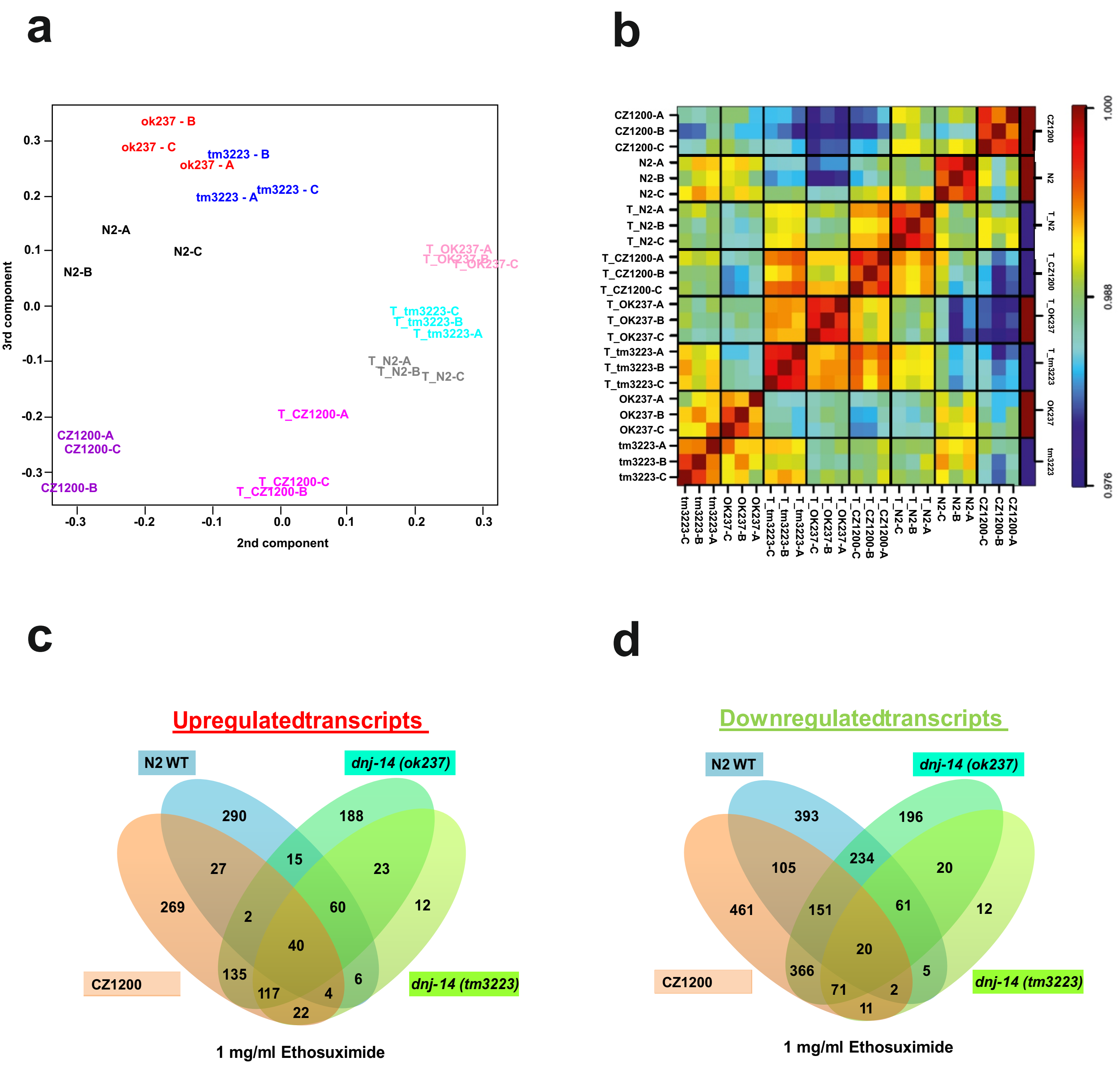

Figure S3. Whole-genome expression profiling of *dnj-14(ok237), dnj-14(tm3223)*, CZ1200 and N2 strains treated with ethosuximide. (A) Principal component analysis of transcription profiles in untreated and ethosuximide treated strains. All three biological replicates in each condition show high reproducibility. (B) Pearson correlation coefficient-based heatmap of expression similarity and divergences among significantly correlated genes among all 24 arrays. High covariance is indicated in dark red, low covariance is indicated in dark blue (see colour bar). C-D) Venn diagrams summarising the level of overlap of significantly up (left)- or down (right)-regulated genes in ethosuximide-treated relative to untreated control group. Numbers of genes uniquely to one of the treated strains or commonly expressed within each subset are listed. Criteria for inclusion was a FDR corrected *p*-value less than 0.01.
