# Supplementary material for: Ethosuximide ameliorates neurodegenerative disease phenotypes by modulating DAF-16/FOXO target gene expression
Source: Mol Neurodegener. 2015 Sep 29;10:51. doi: 10.1186/s13024-015-0046-3 (PMC4587861; doi:10.1186/s13024-015-0046-3)
Supplement: Additional file 6: Figure S4. — Functional enrichment analysis of common DEGs. (PDF 1365 kb) [file 13024_2015_46_MOESM6_ESM.pdf]

a

Functional clustering of up-regulated proteins	
**Cluster**	**Enrichment score**
Oxidation reduction	5.45
Lipid metabolism	4.23
NAD binding and reduction	4.10
Longevity/ageing	3.13
**Functional clustering of down-regulated proteins**	
**Cluster**	**Enrichment score**
Chromatin remodeling	4.29

b

	Gene Ontology Term	Fold enrichment for common DEGs
Biological Process	GO:0055114~oxidation reduction	8.3
	GO:0030258~lipid modification	21.2
	GO:0030259~lipid glycosylation	24.1
	GO:0008340~determination of adult life span	6.1
	GO:0006333~chromatin assembly or disassembly	41.5
	GO:0006334~nucleosome assembly	58.3
	GO:0065004~protein-DNA complex assembly	50.4
	GO:0006323~DNA packaging	44.4
	GO:0051276~chromosome organisation	15.9
	GO:0043933~macromolecular complex subunit organisation	15.4
Cellular Component	GO:0000785~chromatin	39.2
	GO:0032993~protein-DNA complex	54.5
	GO:0005694~chromosome	19.3
Molecular Function	GO:0009055~electron carrier activity	10.5
	GO:0046906~tetrapyrrole binding	11.7
	GO:0005506~iron ion binding	7.4
Protein Domain	IPR017973:cytochrome P450, C-terminal region	28.3
	IPR002213:UDP-glucuronosyl/UDP-glucosyltransferase	22.4
	IPR002347:glucose/ribitol dehydrogenase	26.7
	IPR002198:short-chain dehydrogenase/reductase SDR	20.2
	IPR016040:NAD(P)-binding domain	12.2

c

Publication Enrichment	*p*-Value	Matches
Genes that act downstream of DAF-16 to influence the lifespan of *Caenorhabditis elegans.*	9.96E-05	6

Figure S4. Functional enrichment analysis of common DEGs.

(A) Functional clustering of DEGs that are commonly regulated after ethosuximide treatment. The reported enrichment score was calculated by DAVID according to the Fisher exact score of each clustered term. Only clusters that consisted of 4 or more genes with an enrichment score higher than 1.3 were considered significant and included in the final list of overrepresented GO categories. (B) The fold enrichment for common DEGs in relation to the overall count in significantly enriched GO terms is shown (red = up-regulated, green = down-regulated genes)

(C) modMine publication analysis revealed an enrichment in DAF-16/FOXO regulated genes.
