# Supplementary material for: Ethosuximide ameliorates neurodegenerative disease phenotypes by modulating DAF-16/FOXO target gene expression
Source: Mol Neurodegener. 2015 Sep 29;10:51. doi: 10.1186/s13024-015-0046-3 (PMC4587861; doi:10.1186/s13024-015-0046-3)
Supplement: Additional file 7: Figure S5. — DEGs derived from highly enriched functional clusters. (PDF 33 kb) [file 13024_2015_46_MOESM7_ESM.pdf]

Sequence	Gene	Gene name	Human orthologue
T08B1.3	*alh-5*	ALdehyde deHydrogenase	**ALDH3A2**
C30G12.2	*C30G12.2*	C30G12.2	
K09A11.4	*cyp-14A3*	CYtochrome P450 family	**CYP2A7P1, CYP2J2**
T10H4.11	*cyp-34A2*		**CYP2A7P1**
B0213.15	*cyp-34A9/dod-16*		**CYP2A7P2**
K07C6.4	*cyp-35B1/dod-13*		**CYP2A7P3**
C06B3.3	*cyp-35C1*		**CYP2A7P4**
C12D5.7	*cyp-33A1*		**CYP2J2**
C03G6.15	*cyp-35A2*		**CYP2A7P1**
K09D9.2	*cyp-35A3*		
F55A12.4	*dhs-2*	DeHydrogenases, Short chain	**HSD17B6**
R08H2.1	*dhs-23*		**HSD17B14**
ZK816.5	*dhs-26*		**DHRS1**
F25D1.5	*F25D1.5*		
F08A8.2	*F08A8.2*	F08A8.2	**ACOX1**
C24B9.9	*dod-3*	Downstream Of DAF-16 (regulated by DAF-16)	
T20G5.7	*dod-6*		
H23N18.2	*ugt-14*	UDP-GlucuronosylTransferase	**UGT3A2, UGT3A1**
C10H11.3	*ugt-25*		
F10D2.11	*ugt-41*		
H23N18.3	*ugt-8*		
C03A7.11	*ugt-51*		
C23G10.6	*C23G10.6*		
C54F6.14	*ftn-1*	FerriTiN	**FTH1**
F28D1.3	*thn-1*	THaumatiN family	
T07C4.4	*spp-1*	SaPosin-like Protein family	
Y55B1BR.3	*Y55B1BR.3*	Y55B1BR.3	
C33E10.1	C33E10.1	C33E10.1	
C39B5.2	C39B5.2	C39B5.2	
F45C12.5	*fbxb-11*	F-box B protein	
M01D1.8	*fbxb-41*		
F55C9.13	*fbxb-63*		
Y40B1B.3	*fbxb-66*		
Y73B6BL.9	*hil-2*	HIstone H1 Like	**HIST1H1A**
F22F1.1	*hil-3*		
C01B10.5	*hil-7*		
M163.3	*his-24*	HIStone	
F45F2.12	*his-8*		**HIST1H2BL**
C14F5.1	*dct-1*	DAF-16/FOXO Controlled, germline Tumor affecting	**BNIP3**

Figure S5. DEGs derived from highly enriched functional clusters.

Red shading denotes increased expression, and green shading denotes decreased expression relative to untreated group.
