# Supplementary material for: Ethosuximide ameliorates neurodegenerative disease phenotypes by modulating DAF-16/FOXO target gene expression
Source: Mol Neurodegener. 2015 Sep 29;10:51. doi: 10.1186/s13024-015-0046-3 (PMC4587861; doi:10.1186/s13024-015-0046-3)
Supplement: Additional file 8: Figure S6. — Ethosuximide-responsive genes are enriched for a DAF-16 Associated Element (DAE) motif. (PDF 145 kb) [file 13024_2015_46_MOESM8_ESM.pdf]

Motif	Logo		Occurrences		Other algorithms that identified the regulatory element		
	Consensus	Reverse consensus	Three treated strains	All four treated strains	MEME-DREME	BioProspector	SCOPE
DAF-16 Associated Element (DAE) CTTATCA	wktCTTATCAGtytt 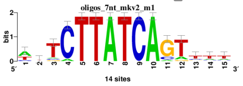	AARACTGATAAGAMW 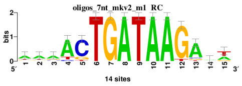	19%	24%	✓	✓	✓
	arraCTGATAAGrawr 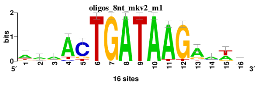	YWTYCTTATCAGTYYT 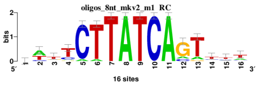	25%	25%	✓	✓	✓
	kwwTCTTATCASts 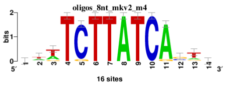	SASTGATAAGAWWM 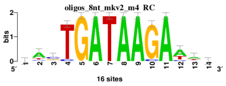	15%	-	X	✓	X
TATA box GTATA(T/A)(T/ A)AG	tRTATAAAkr 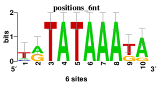	YMTTTATAYA 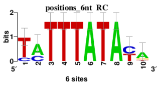	22%	12%	✓	X	✓
	yyCTTTTATAsm 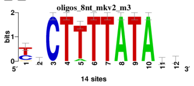	KSTATAAAAGRR 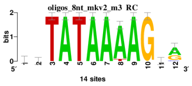	12%	-	X	X	X
?	msACTACAAcct 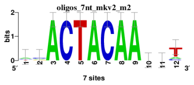	AGGTTGTAGTSK 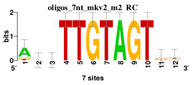	-	15%	X	X	X

Figure S6. Ethosuximide-responsive genes are enriched for a DAF-16 Associated Element (DAE) motif. To identify candidate regulatory sequence motifs for ethosuximide-responsive genes, 200 bp upstream regions for each of promoter sequences of common DEGs were mined for overrepresented motifs using the ‘oligo-analysis’ and ‘peak-motifs analysis’ option of RSAT web server. Repeat analyses were also performed using multiple motif analysing packages. Table shows overrepresented consensus motifs found by RSAT peak-motifs analysis containing a graphic representation of the motifs identified and their reverse complements, along with the extent of agreement with BioProspector, MEME and SCOPE packages.
