# Supplementary material for: Ethosuximide ameliorates neurodegenerative disease phenotypes by modulating DAF-16/FOXO target gene expression
Source: Mol Neurodegener. 2015 Sep 29;10:51. doi: 10.1186/s13024-015-0046-3 (PMC4587861; doi:10.1186/s13024-015-0046-3)
Supplement: Additional file 9: Figure S7. — Data set associations. (PDF 1374 kb) [file 13024_2015_46_MOESM9_ESM.pdf]

a

	Triply regulated			Quadruply regulated		
Rank	Weight	PubMedID	Tag(s)	Weight	PubMedID	Tag(s)
1	3.60%	18778409	Ageing	3.20%	18778409	Ageing
2	1.60%	21589891	Germline	3.00%	21931806	Chemical stimulus
3	1.60%	23352664	Lifespan	2.40%	23300685	Oxidative stress, lifespan
4	1.60%	21931806	Chemical stimulus	1.90%	20798549	Metabolism
5	1.50%	20798549	Metabolism	1.70%	22560298	Gene expression
6	1.50%	19745112	Gene expression	1.70%	20816092	Insulin-like signaling, stress
7	1.40%	15772128	Development	1.60%	19379696	Germline immortality
8	1.40%	21765926	Stress hormesis	1.60%	22083954	Innate immunity
9	1.30%	21363964	Dosage compensation	1.50%	21363964	Dosage compensation
10	1.20%	23300685	Oxidative stress, lifespan	1.10%	21723504	Lifespan, stress response

b

	Gene Array	Triply regulated		Quadruply regulated	
		Up	Down	Up	Down
Longevity mutants and dauers	*daf-2* pathway targets	14%		8%	
	daf-16 pathway targets	6%	4%	5%	2%
	Age-regulated genes	3%		2%	
	Shared dauer and insulin pathway targets	1%	0%	1%	0%
	Most responsive *daf-16* regulated genes	16%	6%	14%	2%
Food deprivation	Genes in response to fasting	0%	11%	0%	11%
	Starvation (dietary restriction)	4%		3%	
Stress	*skn-1* pathway targets	0%		0%	
	Hyperbaric oxygen stress (oxidative stress)	1%	0%	1%	0%
	Commonly regulated in oxidative stress and aging	4%	0%	4%	0%
	Commonly regulated in oxidative stress and *daf-16*	8%	0%	7%	0%
	Commonly regulated in stress, aging, and *daf-16*	4%	0%	10%	0%
Polyphenolic action	1 mM Resveratrol regulated genes	1%		1%	
	200 μM Quercetin regulated genes	2%		1%	
	300 μM Tannic acid regulated genes	0%		0%	
Immune response	Immune effector genes	15%		4%	
	daf-2 pathway and *Pseudomonas aeruginosa* infection	2%		2%	
Toxin	Genes in response to cadmium toxicity	6%	6%	5%	4%

c

Lack of enrichment of known detoxification enzymes						
Enzyme	Total # in Genome	Abbr.	N2 WT	P_{unc-25}::GFP	*dnj-14(ok237)*	*dnj-14(tm3223)*
Cytochrome P450	75	cyp	8	12	17	12
UDP-glucuronosyl/glucosyl transferase	72	ugt	7	8	15	12
Glutathione peroxidase	8	gpx	0	0	0	0
Glutathione S-transferase	57	gst	1	5	5	3
Superoxide dismutase	5	sod	0	0	1	0
Thioredoxin	5	trx	1	1	1	0
Thioredoxin reductase	2	trxr	0	0	0	0
Catalase	3	ctl	0	0	0	0
Peroxiredoxin	3	prdx	0	0	0	0
Sestrin	1	sesn	0	0	0	0
Isocitrate dehydrogenase	2	idh	0	0	0	0
Glutaredoxin	5	glrx	0	0	0	0
Ferritin	2	ftn	1	1	1	1

Figure S7. Data set associations. (A) Top ten data set associations for stringent common DEGs queried using the SPELL search engine. Gene expression data sets and their associated PubMed ID, tags and weight are indicated. (B) Manual comparisons of gene expression patterns of relevant gene clusters or transcriptional profiles in the literature to findings in this study. Only a small proportion of common DEGs overlap with expression profiles of various stress conditions. (C) Lack of enrichment of known detoxification enzymes in response to ethosuximide treatment.
