# Supplementary material for: Ethosuximide ameliorates neurodegenerative disease phenotypes by modulating DAF-16/FOXO target gene expression
Source: Mol Neurodegener. 2015 Sep 29;10:51. doi: 10.1186/s13024-015-0046-3 (PMC4587861; doi:10.1186/s13024-015-0046-3)
Supplement: Additional file 10: Figure S8. — Ethosuximide does not induce general stress and hormesis. (PDF 1626 kb) [file 13024_2015_46_MOESM10_ESM.pdf]

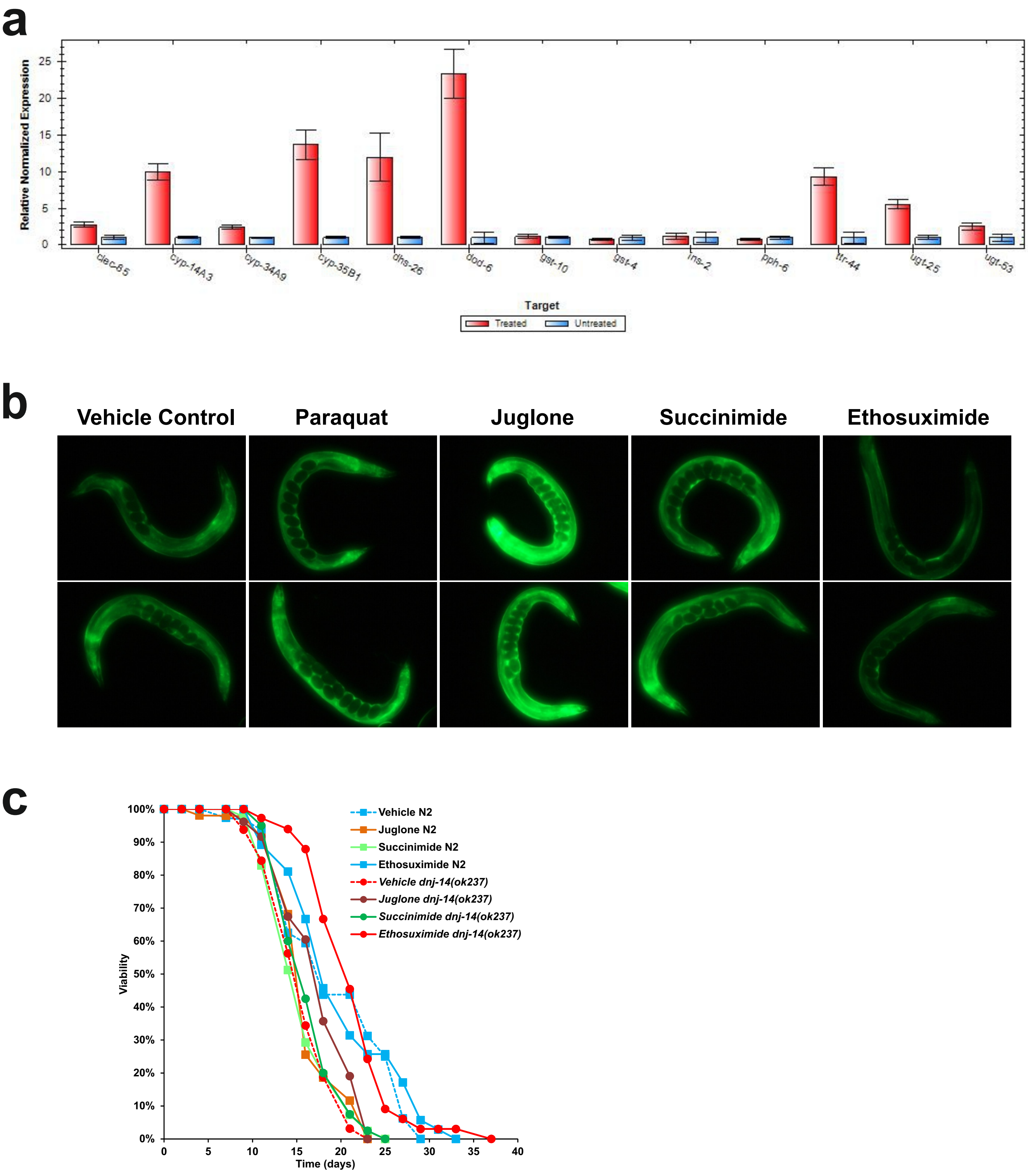

Figure S8. Ethosuximide does not induce general stress and hormesis.

(A) qRT-PCR analysis of the stress response genes *gst-4* and *gst-10* showed no evidence of induction by ethosuximide (B) Ethosuximide does not promote activation of an oxidative stress-responsive gene reporter. Young adult N2 worms carrying a $P_{gst\text{-}4}$::GFP transgene were exposed to various treatments and observed for transgene expression by fluorescence microscopy. Note the lack of $P_{gst\text{-}4}$::GFP induction following ethosuximide exposure in comparison to the oxidative stressors juglone and paraquat (positive transgene induction controls). (C) Lifespan increase by ethosuximide in *dnj-14* mutants is not mimicked by hormetic compounds or the non-therapeutic compound, succinimide.
