# Supplementary material for: Ethosuximide ameliorates neurodegenerative disease phenotypes by modulating DAF-16/FOXO target gene expression
Source: Mol Neurodegener. 2015 Sep 29;10:51. doi: 10.1186/s13024-015-0046-3 (PMC4587861; doi:10.1186/s13024-015-0046-3)
Supplement: Additional file 11: Figure S9. — Negative genetic interactions between dnj-14 and daf-16. (PDF 1580 kb) [file 13024_2015_46_MOESM11_ESM.pdf]

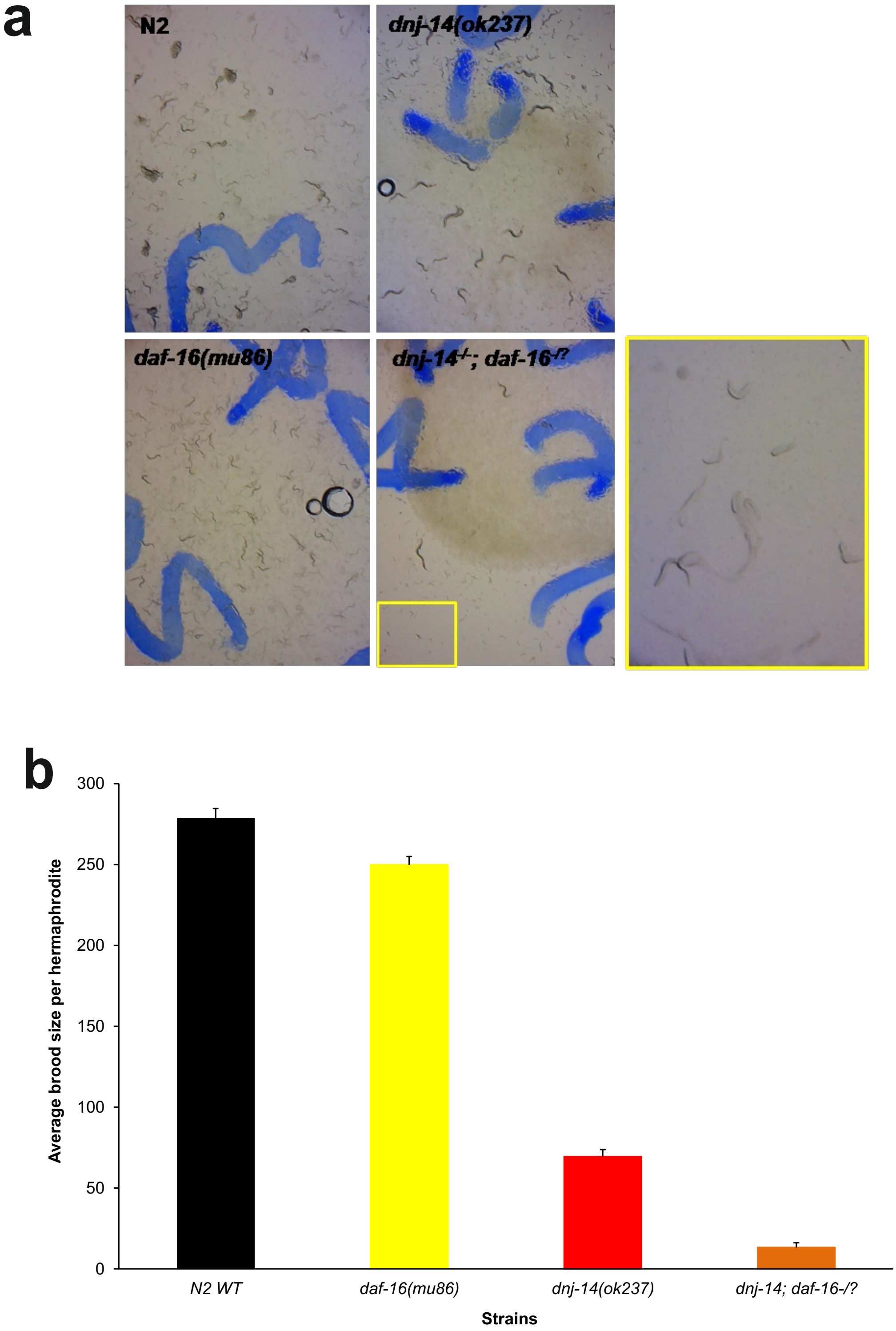

Fig S9: Negative genetic interactions between *dnj-14(ok237)* and *daf-16(mu86)*.

A) Macroscopic images of seeded NGM plates containing putative double mutant (*dnj-14-/-;daf-16-/?*) cross-progeny relative to N2 worms and single mutants are shown. Single plates were initiated with one L3-L4-stage larva and grown for 15 days before imaging the central two-thirds of each plate at equivalent magnification. On the N2 and single mutants' plates, the food supply was exhausted rapidly by the fast growing worms. In contrast, the OP50 lawn remained the same on the putative double mutant plate and many of the progeny arrested at early larval stages (*yellow inset box*). B) Brood sizes were measured by single plating L4 hermaphrodites from each strain and transferring each adult to a fresh plate daily. Progeny were counted immediately after each transfer.
