# Supplementary material for: Ethosuximide ameliorates neurodegenerative disease phenotypes by modulating DAF-16/FOXO target gene expression
Source: Mol Neurodegener. 2015 Sep 29;10:51. doi: 10.1186/s13024-015-0046-3 (PMC4587861; doi:10.1186/s13024-015-0046-3)
Supplement: Additional file 12: Figure S10. — Ethosuximide action is independent of hsp-1. (PDF 98 kb) [file 13024_2015_46_MOESM12_ESM.pdf]

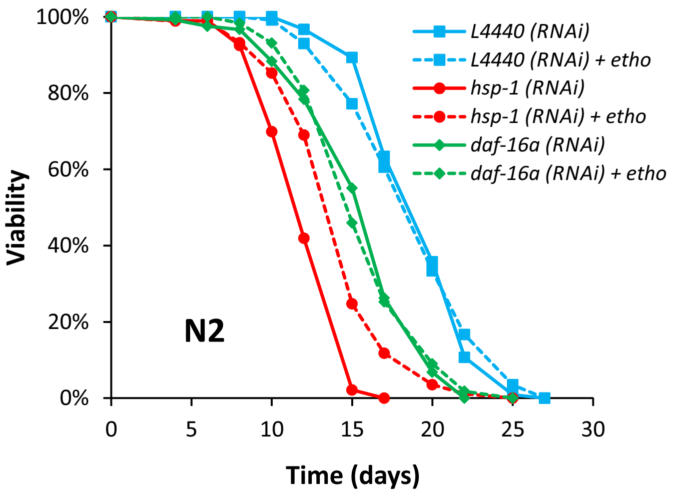

C╩ĵ ’ŕś { 10. Ethosuximide action is independent of *hsp-1*.

Survival curves of wild type N2 worms grown on *E. coli* containing empty vector (L4440), *hsp-1* or *daf-16* dsRNA-producing plasmids in the presence (dashed lines) or absence (solid lines) of 1 mg/ml ethosuximide. RNAi of *hsp-1* and *daf-16* reduced lifespan compared to the vector control. Although ethosuximide treatment significantly increased the lifespan of worms on *hsp-1* RNAi (p<0.001), it had no significant effect on vector control or *daf-16* RNAi worms (p>0.15) (n>100 worms per strain/condition).
