# Supplementary material for: Ethosuximide ameliorates neurodegenerative disease phenotypes by modulating DAF-16/FOXO target gene expression
Source: Mol Neurodegener. 2015 Sep 29;10:51. doi: 10.1186/s13024-015-0046-3 (PMC4587861; doi:10.1186/s13024-015-0046-3)
Supplement: Additional file 13: Figure S11. — Ethosuximide does not cause obvious nuclear translocation of GFP-tagged DAF-16. (PDF 1626 kb) [file 13024_2015_46_MOESM13_ESM.pdf]

a

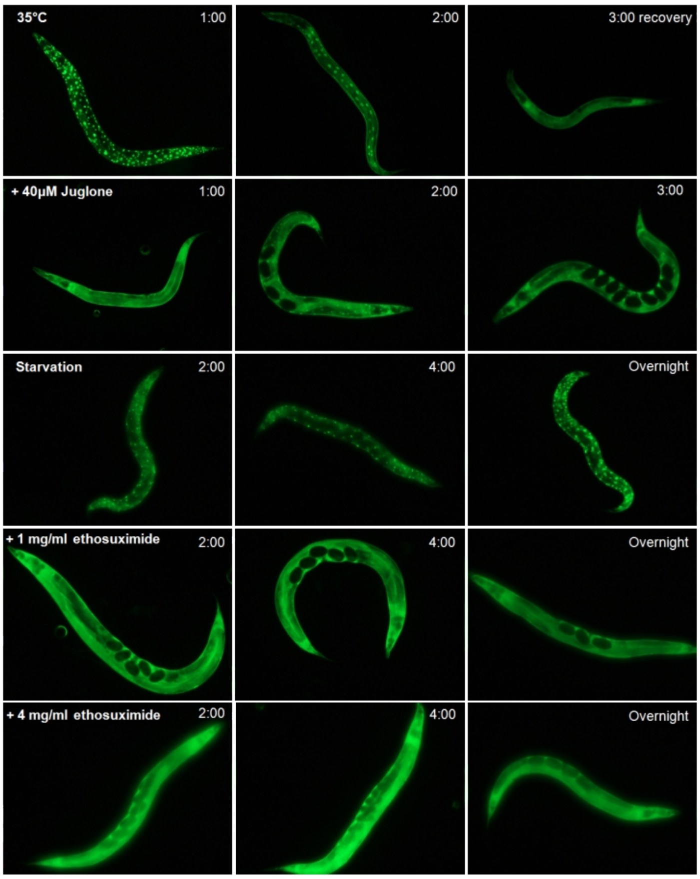

b

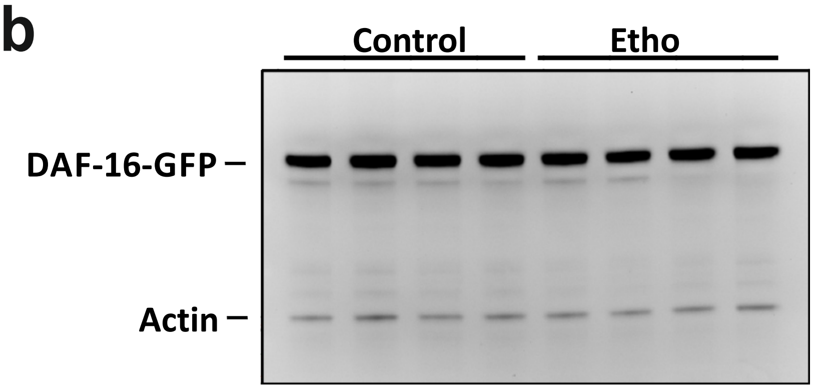

Figure S11: Ethosuximide does not cause obvious nuclear translocation of GFP-tagged DAF-16. The fluorescence pattern and protein levels of DAF-16-GFP were monitored. A) Fluorescent micrographs of *daf-16* translational GFP reporter expression pattern in young TJ356 adults that were subjected to different durations of heat-stress, oxidative-stress (juglone), starvation and 1 or 4 mg/ml ethosuximide . As expected, heat-shocking and overnight starvation gave rise to a dramatic nuclear accumulation of DAF-16-GFP. Upon recovery post-heat shocking, DAF-16-GFP exhibited a largely uniform cytosolic distribution pattern (top right panel). Exposure of worms to 40 µM juglone resulted in subtle DAF-16 nuclear localisation; whereas no obvious nuclear accumulation of DAF-16-GFP was observed in the presence of either 1 or 4 mg/ml ethosuximide. Time after exposure is listed as hours:minutes. Representative images are shown of n = 25 worms examined in each of three independent experiments. B) DAF-16-GFP protein level was also assessed following 1 mg/ml ethosuximide exposure by probing western blots with anti-GFP and anti-actin antibodies.
