# Supplementary material for: Ethosuximide ameliorates neurodegenerative disease phenotypes by modulating DAF-16/FOXO target gene expression
Source: Mol Neurodegener. 2015 Sep 29;10:51. doi: 10.1186/s13024-015-0046-3 (PMC4587861; doi:10.1186/s13024-015-0046-3)
Supplement: Additional file 14: Figure S12. — Ethosuximide reduces polyglutamine protein aggregation in mammalian neurons. (PDF 2095 kb) [file 13024_2015_46_MOESM14_ESM.pdf]

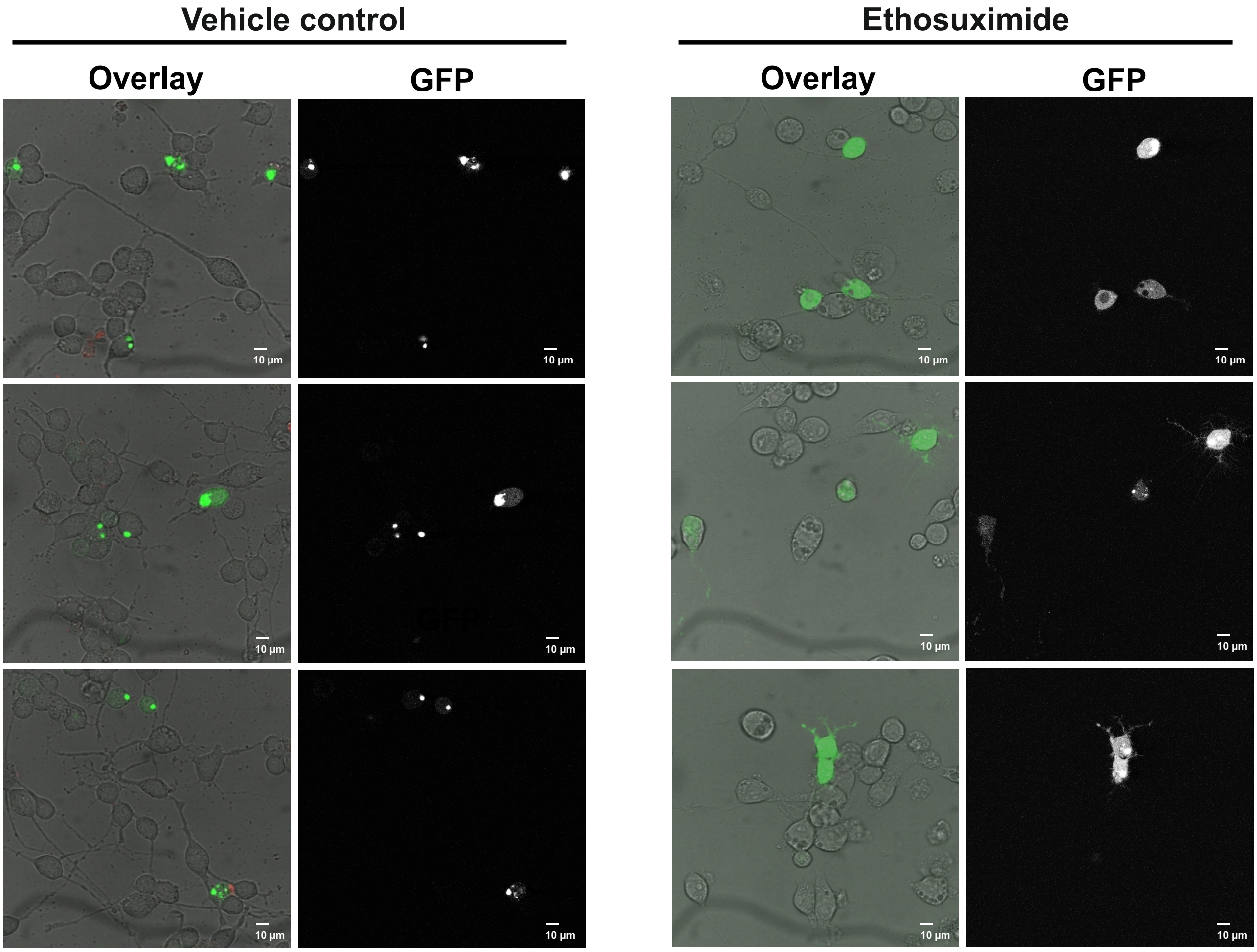

Figure S12. Ethosuximide reduces polyglutamine protein aggregation in mammalian neurons. Visualisation of the effect of 1mg/ml ethosuximide on polyQ97-EGFP aggregation in N2A cells in comparison to vehicle control. All cells were imaged 72 hours post-transfection. Phase contrast, GFP (green) and SYTOX orange staining (red, to identify dead cells) images are overlaid on the left panel, with GFP shown on the right panel. Scale bar = 10um.
