# Supplementary material for: Ethosuximide ameliorates neurodegenerative disease phenotypes by modulating DAF-16/FOXO target gene expression
Source: Mol Neurodegener. 2015 Sep 29;10:51. doi: 10.1186/s13024-015-0046-3 (PMC4587861; doi:10.1186/s13024-015-0046-3)
Supplement: Additional file 15: Figure S13. — Ethosuximide has no significant effect on cytotoxicity in mammalian neurons. (PDF 33 kb) [file 13024_2015_46_MOESM15_ESM.pdf]

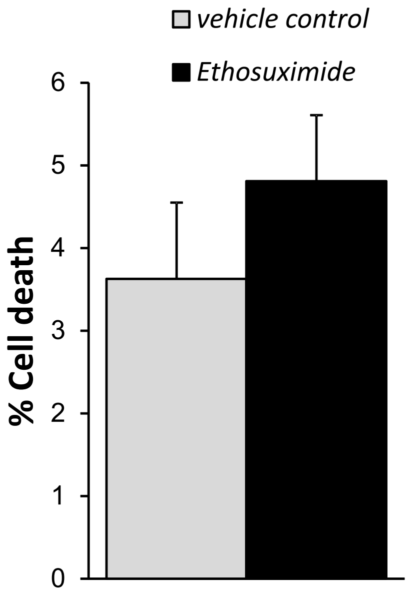

C╨ĵ ’ŕś S13. Ethosuximide has no significant effect on cytotoxicity in mammalian neurons. The number of SYTOX orange-stained **(**dead) cells present 72 hours post-transfection was counted manually and expressed as the percentage of the total number of cells in each microscope field examined. Over 800 cells were counted from three independent experiments. No significant difference was observed between the percentage of dead cells present in cultures incubated with vehicle control or 1mg/ml ethosuximide.
