# Supplementary material for: Ethosuximide ameliorates neurodegenerative disease phenotypes by modulating DAF-16/FOXO target gene expression
Source: Mol Neurodegener. 2015 Sep 29;10:51. doi: 10.1186/s13024-015-0046-3 (PMC4587861; doi:10.1186/s13024-015-0046-3)
Supplement: Additional file 16: Table S2. — C. elegans strains used in this study. (PDF 12 kb) [file 13024_2015_46_MOESM16_ESM.pdf]

Strain	Relevant genotype	Source
N2	*C. elegans* wild type var. Bristol	CGC
RM2754	*dnj-14(ok237)X*	CGC
JD21	*cca-1 (ad1650)X*	CGC
TM3223	*dnj-14(tm3223)X*	S. Mitani
CZ1200	*lin-15(n756)X*; *juIs76*[$P_{unc\text{-}25}$::GFP + *lin-15(+)*]	B.C. Kraemer
CK10	*bkIs10* [$P_{aex\text{-}3}$*::h4R1N TauV337M* + $P_{myo\text{-}2}$*::GFP*]	B.C. Kraemer
AMG119	*cca-1 (ad1650)X; bkIs10* [$P_{aex\text{-}3}$*::h4R1N TauV337M* + $P_{myo\text{-}2}$*::GFP*]	X. Chen

Table S2: *C. elegans* strains used in this study
