# Supplementary material for: Ethosuximide ameliorates neurodegenerative disease phenotypes by modulating DAF-16/FOXO target gene expression
Source: Mol Neurodegener. 2015 Sep 29;10:51. doi: 10.1186/s13024-015-0046-3 (PMC4587861; doi:10.1186/s13024-015-0046-3)
Supplement: Additional file 17: — Supplementary Methods. (PDF 70 kb) [file 13024_2015_46_MOESM17_ESM.pdf]

SUPPLEMENTARY MATERIALS AND METHODS

PROTEIN WORK

Sequential extraction of Tau protein

Worm lysates were prepared following growth of mixed stage populations of vehicle and ethosuximide treated Tau V337M worms to near-confluency on twenty 90 mm diameter NGM plates. Worms were washed off plates with M9 buffer and the collected worm pellets were then stored at -80°C. After the freeze thaw cycle, frozen animals were directly homogenised using a Mikro-Dismembrator (B. Braun Biotech) at 2000 rpm for 2 minutes in an equal volume of acid-washed glass beads (425–600 µm; Sigma) and high-salt reassembly (RAB) buffer (100 mM MES, 1 mM EGTA, 0.5 mM $MgSO_4$, 20 mM NaF, 0.75 M NaCl, 0.5 mM PMSF, 0.1% protease inhibitor cocktail, pH 7.0). These homogenates were centrifuged at 5000x*g* for 5 minutes at 4°C to obtain crude lysates, which were used to quantify total protein levels by western blotting. The lysates were then ultracentrifuged at 40,000x*g* at 4°C for 40 minutes. The supernatant was subsequently boiled for 5 minutes, and the sample was centrifuged at 13,000x*g* at 4°C for 15 min. This supernatant constitutes the RAB fraction containing normal, soluble Tau. The RAB-insoluble material was re-homogenized with radio-immunoprecipitation assay (RIPA) buffer (150 mM NaCl, 1% Nonidet P-40, 0.5% deoxycholate, 5 mM EDTA, 0.1% SDS, 50 mM Tris, 0.5 mM PMSF, 0.1% protease inhibitor cocktail, pH 8.0) by sonication and

centrifuged for 20 min at 40,000x*g* to remove membrane proteins. The resulting supernatant is the RIPA fraction containing abnormal, detergent-solubilised Tau.

TRANSCRIPTOMIC WORK

C. elegans growth and collection

We first bleached worms to synchronise embryos and these were then hatched on ethosuximide plates and grown at 20°C until day 2 of adulthood. 50 gravid hermaphrodites of each strain were then picked to lay eggs on one drug dish for six hours at room temperature. Progeny were grown and 400 were selected at the L4 stage and transferred every 2 days until day 6 of adulthood was reached. 40 hermaphrodites were cultured on each Petri dish. When worms reached age day 6, they were washed off plates using 100 µl DNase/RNase-free distilled water (Invitrogen) and 400 µl of TRIzol® Reagent (Invitrogen) was added and the mixture was vortexed for 2 minutes. Worms were stored at -80°C until RNA isolation. Four individual biological replicates were prepared for each strain/treatment.

RNA extraction and purification

Total RNA was extracted using a combined TRIzol® Reagent (Invitrogen) and RNAeasy (Qiagen) column method. Following one freeze and thaw cycle, samples were disrupted and homogenised on a vortex shaker in a 4°C cold room for 40 minutes. A further 200 µl of TRIzol® was added and vortexed before the

second freeze and thaw cycle. 140 µl of chloroform was added before centrifugation at 12,000x*g* for 15 minutes at 4°C. Following two separations of aqueous upper phase with chloroform, RNA was precipitated by adding dropwise to an equal volume of 70% ethanol made in RNase-free water. RNA was then treated with RNase-free DNase I (Qiagen) before purification and concentration using RNeasy Mini Kit (Qiagen) according to manufacturer's instructions. RNA concentration and purity were quantified initially using a NanoDrop 1000 spectrophotometer (Thermo Fisher Scientific, Waltham, MA, USA) and RNA quality was subsequently ascertained using an Agilent 2100 Bioanalyser RNA 6000. An RNA integrity number (RIN) between 1 and 10 was then assigned, where 1 = degraded and 10 = intact. In this study, only RINs of greater than 7 out of 10 were accepted for further analysis and all RNA samples submitted showed no signs of degradation. The best three preparations per condition were used for subsequent RNA amplification, labelling and hybridisation to Affymetrix *C. elegans* GeneChips (Affymetrix, High Wycombe, UK). The remaining preparations were kept for qPCR validation of microarray results. Purified total RNA samples free from genomic DNA were then labelled using an Affymetrix GeneChip 3'IVT Express Kit. A two-cycle target labelling protocol was followed using 100ng of input RNA. cDNA was synthesized in the first cycle followed by in vitro transcription (IVT) amplification with an unlabelled ribonucleotide mix. This was followed by a second cycle of cDNA synthesis and the double stranded cDNA was then amplified and labelled using a biotinylated ribonucleotide mix in the second IVT reaction. This labelled cRNA was then cleaned up, fragmented

and hybridised to GeneChip expression arrays for 16 hours at 45°C in a GeneChip Hybridisation Oven 640. Following hybridisation the arrays were washed on a GeneChip® Fluidics Station 450 using the GeneChip Hybridisation Wash and Stain kit and fluidics script FS450_0004 and scanned using the GeneChip® Scanner 3000 7G.

Data generation and diagnostic analysis

Initial diagnostic and differential expression (DE) analysis of the microarray data was performed using the statistical analysis tools R, Affy (version 2.15.2) and limma (version 3.14.4). Background correction and normalisation of raw Affymetrix array data were conducted using the Robust Multi-array Average (RMA) function built into the Affy package(*2*). The significance test of the estimated logFC (log_2 Fold Change) for each contrast was performed by the empirical Bayes function packed in limma and *p*-values were adjusted using the Benjamini and Hochberg False discovery rate (FDR) control approach to deal with the effect of multiple tests(*3*). DE genes (DEGs) with a FDR corrected *p*-value less or equal to than 0.01 and fold change greater than 2 were deemed to be significantly differentially expressed.

Gene Ontology analysis

The functional annotation and clustering of putative ethosuximide targets were assessed using both the Database for Annotation, Visualization and Integrated Discovery (DAVID) v6.7 (http://david.abcc.ncifcrf.gov/) (*4*) and modENCODE

Modmine bioinformatic suites (http://intermine.modencode.org/) (*5*) in order to identify trends in the biological processes and molecular functions of the identified DEGs. For gene enrichment analysis, Affymetrix *C. elegans* probe IDs of commonly regulated DEGs were first submitted to DAVID as a “Gene list” and following selection of *C. elegans* background, were converted to either Ensembl or Worm Base IDs using the Gene ID Conversion Tool (>90% converted). For GO Term analysis, the “Biological Process”, “Cellular Component” and “Molecular Function” categories were studied using the GO FAT default settings. Functional annotation clustering was performed with the default criteria and prevalence of annotation terms within the list of DE genes was compared to the prevalence in the whole *C. elegans* genome. Protein domains and pathway enrichment were also determined using DAVID by querying the Interpro and Kyoto Encyclopedia of Genes and Genomes (KEGG) databases, respectively. Enriched genes were obtained by performing Gene Functional Classification Clustering using threshold count 5, EASE 0.1 and medium stringency. GO terms and domains with less than 4 genes were discarded and an enrichment score over 1.3 represents a significant enrichment. Enrichment in GO categories, protein domains, pathways and publications were also assessed using modENCODE modMine. Human orthologues of the corresponding worm genes were derived from the OrthoList online tool (http://www.greenwaldlab.org/ortholist.). Within the search window, the list of enriched *C. elegans* DEGs was inputted as Ensembl gene IDs to retrieve the predicted human genes which were in the Ensembl ENSG ID format. All

orthologous genes with a duplicate output in one of the species were counted only as one gene.

Motif analysis

Using complementary algorithms, we analysed within up to 200 bp of intergenic sequence upstream of common DEGs to identify shared DNA motifs. Regulatory Sequence Analysis Tools (RSAT; http://rsat.bigre.ulb.ac.be/rsat/) was first used to retrieve the 200 bp upstream sequences of *C. elegans* genes, which were then submitted to RSAT oligo analysis and peak-motifs analysis, multiple expectation maximisation for motif elicitation (MEME, http://meme.nbcr.net/meme/), BioProspector (http://seqmotifs.stanford.edu) and SCOPE motif finder (http://genie.dartmouth.edu/scope/), in each case specifying an oligonucleotide length of 6-8 bases while all the other parameters were set as default, to identify sequence elements that were statistically overrepresented. To display consensus motifs generated from BioProspector, retrieved motif matrices in TRANSFAC format were analysed by WebLogo (http://weblogo.berkeley.edu/logo.cgi).

Quantitative real-time PCR (qRT-PCR)

qRT-PCR primers (listed in Table S3) were designed using the DNA Dynamo Sequence Analysis Software (Blue Tractor Software, Llanfairfechan, UK) to the following criteria: gene specific primers were all between 20 and 25 bp long; the product of the PCR was between 280-330 bp in length; the product spanned an intron to give differentially sized genomic and cDNA products; the melting

temperatures of the primer pairs were matched and were between 55 and 60°C. *act-1* and *pmp-3* which showed no significant changes in microarray analysis and have been extensively used as normalising genes in differential expression studies in *C. elegans* were chosen as reference genes. The compatibility and specificity of these primers were tested on samples from the microarray, and analysed by gel electrophoresis. Only primer sets showing single bands of the expected size were used for qPCR analysis. Melting curve analysis during qPCR was also performed with each primer pair to ensure that quantification is the result of only one product.

For each synthesis reaction, appropriate RNA sample volume was added to ensure the presence of 200 ng of total RNA for each sample in the group, and 1 µl of random primers (250 ng/µl; Promega), 1 µl of dNTPs (10 mM; Invitrogen), and RNase-free water were added up to a volume of 12 µl. Samples were mixed, centrifuged briefly and incubated for 5 minutes at 70°C, and then immediately chilled on ice for 2 minutes. After this first incubation, 4 µl of 5X M-MuLV RNaseH- Reaction buffer for RT, 2 µl of 0.1 M DTT, 1 µl of RNaseOUT (40U/µl) and 1 µl of ProtoScript M-MuLV Reverse Transcriptase (NEB) were added to the 12 µl mix as a master mix. Contents were then incubated in a G-Storm GSX thermal cycler (GRI, Braintree, UK) first at 25°C for 5 minutes followed by 1 hour at 42°C and finally 2 minutes at 92°C to inactivate the RT enzyme. The cDNA was diluted (1:50) with DEPC treated water and stored at −80°C until required.

For gene expression analysis, master mixes for diluted cDNA samples mixed with DEPC water and selected qRT-CPR primers mixed with iTaq™ Universal

SYBR® Green Supermix (BioRad) were made and added to 96-well plates sequentially to make a final reaction volume of 10 µl per well. A non-template control (sample substituted by sterile water) was also included. Reactions were performed using an IQ5 real-time PCR detection system (Bio-Rad). The thermocycler was programmed to first heat to 95°C for 3 minutes, followed by 40 cycles of a 10 seconds denaturation step at 95ºC, a 30 seconds annealing step at 58°C, and a 30 seconds elongation step at 70ºC. The final cycle consisted of 30 seconds at 95ºC and after the thermocycling reaction, a 65-95°C melt curve with a gradient of 0.5°C was inserted, allowing detection of possible nonspecific products. All qRT-PCR reactions were carried out on 3 biological replicates with three technical replications and transcript expression was analysed using Bio-Rad CFX Manager 3.0 software. The relative expression of each gene was calculated by normalising the Ct values for test genes to the two reference genes (*act-1* and *pmp-3*).

MAMMALIAN CELL WORK

Cell culture

Mouse Neuro2A (N2A) neuroblastoma cells were passaged every 2-5 days. Adherent cells were maintained as a monolayer culture in 75 cm^2 culture flasks in Dulbecco's Modified Eagle Medium (DMEM Gibco) supplemented with 10% foetal bovine serum (FBS), 1% penicillin/streptomycin and 1% non-essential amino acids (Gibco) and maintained in 75 cm^2 flasks at 37°C in a humidified atmosphere of 95%air/5% CO_2. Cells were subcultured at or near confluency by

trypsinisation (0.25% trypsin; Mediatech Inc.) followed by dispersion of the cells into single cell suspension in new growth medium and plating onto new growing surfaces. Cell counts were assessed by applying an equal mixture of trypan blue dye and cell suspensions to the chambers of a haemocytometer. For qRT-PCR analysis, cells were grown on 6-well plates. For microscopy, cells were seeded on glass-bottom 35 mm dishes (10 mm glass surface diameter, MatTek Corporation, Ashland, MA, USA).

Transfection

Cells were taken at 60-80% confluence and plated out in 35-mm glass bottom dishes (MatTek, Ashland, MA). To stimulate differentiation prior to transient transfection and to limit the loss of compound activity as a result of protein binding in the serum-cultured cell lines, N2A cell media was exchanged for DMEM supplemented with 2% FBS, 1% penicillin-streptomycin, 20 µM retinoic acid and 0.56 mg/ml ethosuximide. In all the experiments, cells in medium with equal volumes of PBS served as the controls. Cells were then transiently transfected with 3 µg of polyQ97-eGFP-pcDNA3.1(+) Myc His plasmid constructs or polyQ25-eGFP-pcDNA3.1(+) Myc His control plasmid construct using PromoFectin transfection reagent (PromoKine) according to the manufacturer's protocol. The transfection reaction mixture was incubated at room temperature for 30 minutes to allow the lipid and DNA complexes to associate then added dropwise to the cells. Cells were then maintained at 37°C before being used in experiments.

Ethosuximide treatment and RNA extraction

To analyse the effect of ethosuximide on the expression of FOXO targeted genes, qRT-PCR was used. Cells were plated subconfluently at a density of 1.5×10^6 cells per well in flat bottom 6-well microtiter plates in a total volume of 3 ml growth medium supplemented with 10% regular FBS. After overnight incubation to allow cells to adhere to wells, media was exchanged for DMEM containing 2% FBS, 1% penicillin/streptomycin and 20 µM retinoic acid to induce N2A cell differentiation. After 24 hours, N2A cells were treated with vehicle control (PBS) or increasing concentrations of ethosuximide (100 µg/ml, 0.56 mg/ml and 1 mg/ml, covering the clinically relevant concentrations) for 5 hours. A stock solution of ethosuximide was prepared in PBS at a concentration of 100 mg/ml. Following treatment, the full content of a 6-well culture plate was pooled for each experimental condition. Total RNA was then isolated from 3 independent treatments of N2A cells by first homogenising cells directly in RLT buffer (Qiagen) containing β-merceptoethanol and guanidine and subsequently using RNeasy Mini Kit (Qiagen) following manufacturer's instructions. To avoid DNA contamination, the samples were exposed to an on-column treatment with RNase-Free DNase 1 (Qiagen). The isolated total RNA were quantified by use of OD measurement at 260 and 280 nm using a spectrophotometer (Thermo Fisher Scientific, Madison, WI, USA) (all RNA samples had an A_{280}/A_{260} ratio >1.8).

cDNA synthesis and qRT-PCR quantification of mRNA expression of FOXO target genes

1 μg of DNase-treated total RNA was reverse transcribed to cDNA and qRT-PCR was run as described above. Mouse gene-specific primer sequences obtained from PrimerBank (http://pga.mgh.harvard.edu/primerbank/) were validated by RT-PCR and gel electrophoresis. For each sample, the Ct values for test genes were normalised to that for the reference genes glyceraldehyde-3-phosphate dehydrogenase (GAPDH) and β-actin (ACTB), and a sample without cDNA was used as no template control. Transcript expression analysis was performed with Bio-Rad CFX Manager 3.0 software (Biorad), which automatically set the baseline and the threshold.

Confocal laser scanning microscopy and image analysis

Live transfected cells were imaged using a Leica AOBS SP2 microscope (Leica Microsystems, Heidelberg, Germany) with an optimal pin hole of 1 airy unit and using both a 20x dry objective with a 0.5 numerical aperture and a 63x water-immersion objective with a 1.2 numerical aperture. The percentages of aggregates and rate of cell death were determined at 24, 48 and 72 hours post-transfection. To monitor neuronal viability, media was aspirated before cells were incubated for 20 minutes in 2 ml of Krebs-Ringer buffer [20 mM Hepes (pH 7.4), 145 mM NaCl, 10 mM glucose, 5 mM KCl, 3 mM $CaCl_2$, 1.3 mM $MgCl_2$ and 1.2 mM NaH_2PO_4] containing 0.5 μM SYTOX Orange® nucleic acid dye (Invitrogen)

at 37°C and 5% CO_2. For dual imaging of EGFP-tagged polyQ proteins and dying/non-viable cells labelled with SYTOX Orange®, cells were excited with 488 nm and 453 nm lasers and light was collected at 465-500 nm (Alexa 488) and 615-690 nm (Alexa 594), respectively. The channels of the images were taken sequentially with a frame average of 1. Five to ten low- and high-magnification fields were randomly taken for each condition. EGFP and SYTOX Orange® fluorescence and phase images were exported as Tiff files for analysis and rendering using the Leica LAS AF Lite imaging software. Counting of cells/aggregates was mainly performed manually, but if cells were sufficiently sparse to enable accurate automated quantification, then the ImageJ Java-based imaging software (NIH, USA) was used, in which GFP channel images were measured with the '*Analyze particles*' command with a threshold of 180-255. The average percentage of transfected cells with aggregates was calculated from three independent experiments. Counting of SYTOX-positive cells from the total cell population was performed manually.
