# Supplementary material for: Ethosuximide ameliorates neurodegenerative disease phenotypes by modulating DAF-16/FOXO target gene expression
Source: Mol Neurodegener. 2015 Sep 29;10:51. doi: 10.1186/s13024-015-0046-3 (PMC4587861; doi:10.1186/s13024-015-0046-3)
Supplement: Additional file 18: Table S3. — Sequences of oligonucleotides used in this study. (PDF 27 kb) [file 13024_2015_46_MOESM18_ESM.pdf]

Oligo Name	Sequence 5' to 3'	Amplicon Size (bp)	Purpose
**Nematode**			
pmp-3 F	TGGTGTCGCGATTACTGTAG	283	qRT-PCR
pmp-3 R	GATTTGTTGTCGCAGAGTGG		
act-1 F	CTACGAACTTCCTGACGGACAAG	102	qRT-PCR
act-1 R	CCGGCGGACTCCATACC		
cyp-34A9 F	CGAGACTCTTGCCGTAGACC	149	qRT-PCR
cyp-34A9 R	TTCCACCGGTAACCTCTGTC		
cyp-34A9 F	TAACCGATAGGCCAAAGACG	281	qRT-PCR
cyp-34A9 R	ACAAGATCTCTTCCCGATGC		
cyp-35B1 F	CAAAGATGGAGCAGGAGAGG	131	qRT-PCR
cyp-35B1 R	ATTGAATCCTGCGACCAAAG		
cyp-14A3 F	CAAAGGACCGTTACCATTGC	283	qRT-PCR
cyp-14A3 R	TTGAAGCCTCCAGTTGTCTC		
ugt-25 F	ATGACTCACGGAGGTCTTGG	108	qRT-PCR
ugt-25 R	TGCAAGCATATTCGCATTTC		
gst-4 F	TGCTCAATGTGCCTTACGAG	173	qRT-PCR
gst-4 R	AGTTTTTCCAGCGAGTCCAA		
gst-10 F	AAGAGATTGTGCAGACTGGAG	99	qRT-PCR
gst-10 R	AGAACATGTCGAGGAAGGTTG		
clec-85 F	CCTGATGATAAGTATATTGGAGACCTGT GCTACTC	94	qRT-PCR
clec-85 R	GGTTTTGGCTGTAGCACGCCGACTGAGC ATCC		
ttr-44 F	GACGTCTGATCTGTGGAGAC	301	qRT-PCR
ttr-44 R	TCCAGGTTGAGAACTCCAAG		
dhs-26 F	AACATCTGCAGGATCTTGGG	297	qRT-PCR
dhs-26 R	CACAAAGTGATTGCCCATCC		
dod-6 F	TCCTCGTCATCATGTCTGTC	148	qRT-PCR
dod-6 R	CACTTTCCGCAAGTCTTTGG		
ugt-53 F	AACTTCCCTTTACCCCGAAC	276	qRT-PCR
ugt-53 R	TGTTTCGCTTTACGAATCGC		
ins-2 F	TCTGTCTCCTCTTCACAACTG	203	qRT-PCR
ins-2 R	ACATTCTCCACATGTTGCAAGC		
pph-6 F	ACATGGAGGACTTTCACCTG	307	qRT-PCR
pph-6 R	ATAGCAGTAGTTTGGAGCCG		
dnj-14 (ok237) Full F	GAAAATTGGTGATGATGCTGCAGG	WT 2590, *ok237* 361	*ok237* genotyping PCR
dnj-14 (ok237) Mid F	CGCCAATATCTCCAGTCTCCAATG	WT 1736, *ok237* none	
dnj-14(ok237) R	GTCTCCCAGAGGCAGTCGAACAAC		
cca-1(ad1650) Avery F	CCGCAATTTGCCCTCCACAT	WT 3310, *ad1650* 930	*ad1650* genotyping PCR
cca-1(ad1650) Avery R	ATGAGGATGGCGAAGAGGACC		
cca-1(ad1650) F	CGGAAATATTAACTGTCATTCTGGA	WT 1350, *ad1650* none	*ad1650* homozygotes genotyping
cca-1(ad1650) R	CGTGCCTTCACAACCGAACTG		

Oligo Name	Sequence 5' to 3'	Amplicon Size (bp)	Purpose
Tau F	CAAGCTCGCATGGTCAGTAA	425	Tau genotyping
Tau R	TTCTCAGTGGAGCCGATCTT		
**Mouse**			
Ccng2 F	AGGGGTTCAGCTTTTCGGATT	112	qRT-PCR
Ccng2 R	AGTGTTATCATTCTCCGGGGTAG		
Cdkn1a F	CCTGGTGATGTCCGACCTG	103	qRT-PCR
Cdkn1a R	CCATGAGCGCATCGCAATC		
Cdkn1b F	TCAAACGTGAGAGTGTCTAACG	103	qRT-PCR
Cdkn1b R	CCGGGCCGAAGAGATTTCTG		
Gadd45a F	AGACCCCGATAACGTGGTACT	318	qRT-PCR
Gadd45a R	TGATCCATGTAGCGACTTTCC		
Sod2 F	CAGACCTGCCTTACGACTATGG	113	qRT-PCR
Sod2 R	CTCGGTGGCGTTGAGATTGTT		
Rbl2 F	AACTTCCCCATGATTAGCGATG	97	qRT-PCR
Rbl2 R	GGTTAGAACACTGAAGGGCATTT		
Cat F	GCGTCCAGTGCGCTGTAGA	199	qRT-PCR
Cat R	TCAGGGTGGACGTCAGTGAA		
Eif4ebp1 F	GGGGACTACAGCACCACTC	171	qRT-PCR
Eif4ebp1 R	CTCATCGCTGGTAGGGCTA		
Bcl2l11 F	CCCGGAGATACGGATTGCAC	96	qRT-PCR
Bcl2l11 R	GCCTCGCGGTAATCATTTGC		
Gapdh F	AGGTCGGTGTGAACGGATTTG	123	qRT-PCR
Gapdh R	TGTAGACCATGTAGTTGAGGTCA		
Actb F	GGCTGTATTCCCCTCCATCG	154	qRT-PCR
Actb R	CCAGTTGGTAACAATGCCATGT		

Table S3: Sequences of oligonucleotides used in this study
